# Angstrom‐Scale Confined Ion Sieve and Accelerator for Efficient Aqueous Zinc Batteries

**DOI:** 10.1002/adma.74289

**Published:** 2026-07-29

**Authors:** Xing Peng, Caichao Ye, Yingqiang Li, Zhihang Liu, Kunxi Luan, Jinbo Fan, Honglan Huang, Chao Liu, Na Shen, Zhen Hou, Wenyao Zhang, Yongsheng Fu, Mingzhe Chen, Linfeng Hu, Pan Xiong, Guoxiu Wang, Junwu Zhu

**Affiliations:** ^1^ School of Chemistry and Chemical Engineering Key Laboratory for Soft Chemistry and Functional Materials of Ministry of Education Nanjing University of Science and Technology Nanjing China; ^2^ Academy For Advanced Interdisciplinary Studies & Department of Materials Science and Engineering Guangdong Provincial Key Laboratory of Computational Science and Material Design Southern University of Science and Technology Shenzhen China; ^3^ Centre For Clean Energy Technology School of Mathematical and Physical Sciences Faculty of Science University of Technology Sydney Sydney New South Wales Australia; ^4^ School of Energy and Power Engineering Nanjing University of Science and Technology Nanjing China; ^5^ School of Materials Science and Engineering Southeast University Nanjing China

**Keywords:** angstrom‐scale confined ion accelerator, angstrom‐scale interlayer free spacing, atomic Ti vacancies, Ti_0.87_O_2_ nanosheet, Zn anodes

## Abstract

The commercialization of aqueous zinc–metal batteries (AZMBs) is hindered by dendrite growth caused by uncontrolled Zn^2+^ transport and side reactions involving water and anions. Here, an angstrom–scale confined ion sieve and accelerator is designed using unilamellar Ti_0.87_O_2_ nanosheets with atomic Ti vacancies (∼3.0 × 3.8 Å) and interlayer spacing (∼3.5 Å) to enable selective Zn^2+^ (∼1.5 Å) transport while blocking H_2_O (∼4.0 Å) and SO_4_^2−^ (∼5.9 Å). This design facilitates selective Zn^2+^ ion transport with high flux and Zn/SO_4_^2−^ selectivity, effectively mitigating water–/anion–induced parasitic reactions at the Zn anode. Consequently, the Ti_0.87_O_2_@Zn anode exhibits significantly suppressed dendrite growth and parasitic side reactions during repeated Zn plating/stripping, with stable cycle lives exceeding 5000 and 4000 h at 1 and 5 mA cm^−2^, respectively. The Ah–level Ti_0.87_O_2_@Zn//VO_2_ pouch cell retains 85.4% of its initial capacity after 300 cycles at 3 A g^−1^. This strategy provides a promising interfacial design concept for improving the reversibility of aqueous Zn metal anodes and may inspire the rational design of confined ion–transport interphases for related aqueous battery systems.

## Graphical abstract

An angstrom‐scale confined ion accelerator strategy is proposed for ultra‐stable aqueous Zn anodes. Selective and precise transport of hydrated Zn(H_2_O)_6_^2+^, Zn^2+^ cations, H_2_O molecules, and SO_4_^2−^ anions is achieved by in‐plane atomic Ti vacancies and angstrom‐scale interlayer free spacing within the Ti_0.87_O_2_ interfacial layer, leading to homogeneous Zn deposition and suppressed water‐ and anion‐involved side reactions.

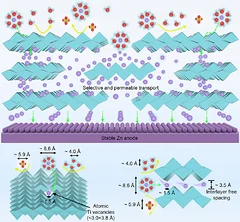

## 1 Introduction

Rechargeable aqueous zinc metal batteries (AZMBs) with aqueous electrolytes have garnered significant attention as a promising alternative to lithium‐ion batteries (LIBs) due to their inherent safety, economic viability, and eco‐friendliness [1, 2]. However, persistent side reactions induced by water and anions, along with uncontrollable dendrite growth on the zinc anode, significantly hinder the commercialization of AZMBs [3, 4]. Unlike the organic electrolytes used in LIBs, aqueous electrolytes with a narrow voltage window contain highly polar water molecules, which easily participate in the solvation sheath structure of Zn^2+^ ions and cause persistent parasitic reactions (e.g., corrosion, hydrogen evolution, and passivation) on the zinc anode [5, 6]. These reactions exacerbate dendritic growth and electrolyte degradation, leading to lower Coulombic efficiency (CE) and a shortened cycle life of the zinc anode [7]. The primary goal for achieving a zinc anode with high stability and prolonged cycling life is to effectively reduce the access of water molecules and anions to the Zn surface thereby suppressing side reactions at the electrolyte/zinc interface, while facilitating uniform and rapid transport of Zn^2+^ ions.

**FIGURE 1:**
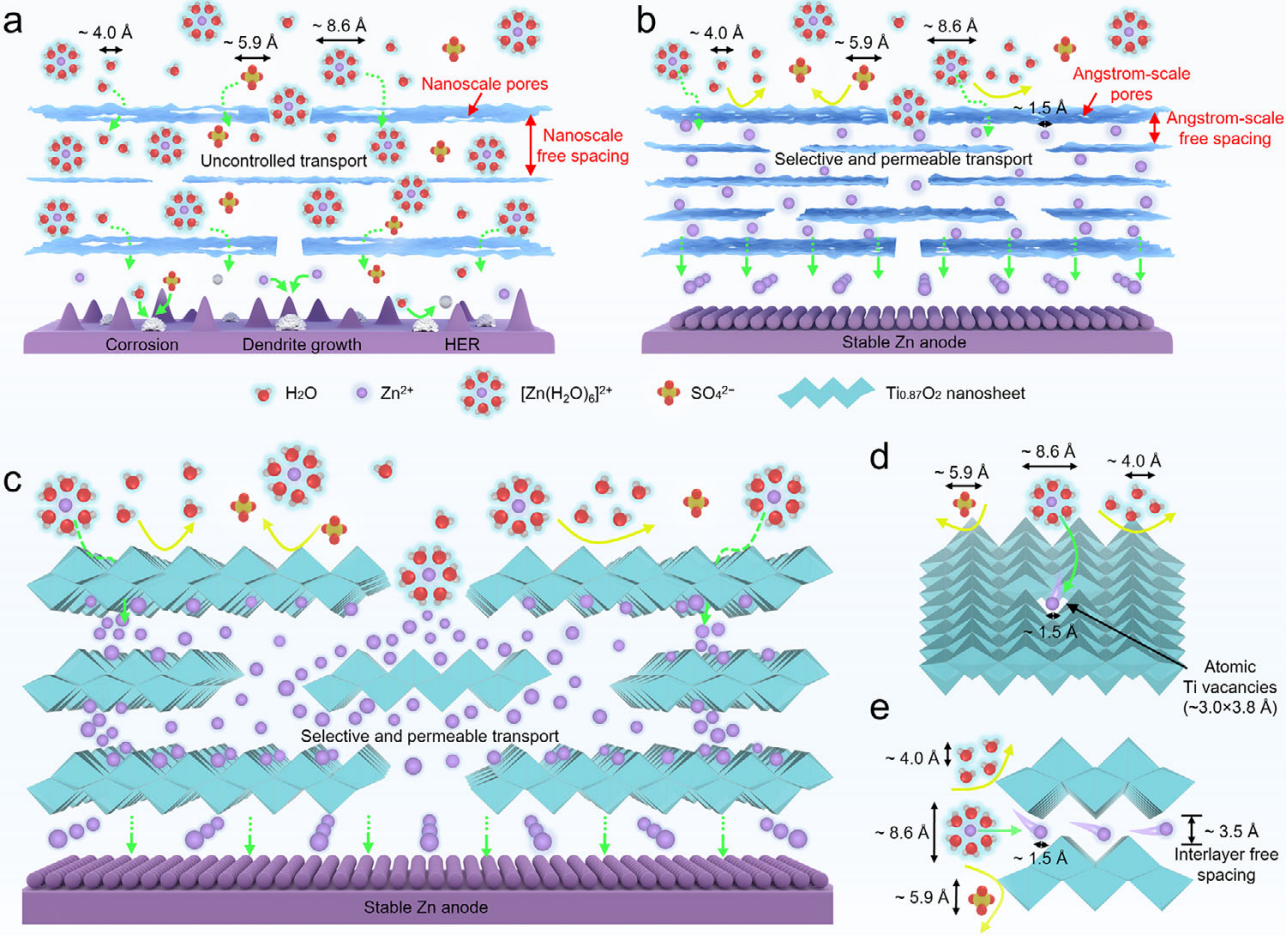
Variation in ion transport mechanisms due to different pore sizes and interlayer spacings, and the structure and mechanism of Ti_0.87_O_2_ nanosheets for stabilizing Zn anodes. (a) Schematic of materials with nanoscale pores and interlayer‐free spacing for stabilizing zinc anodes. (b) Schematic of materials with angstrom‐scale confined channels (>1.5 Å but <4 Å) for stabilizing zinc anodes. The green arrow indicates the free passage of ions or molecules, while the yellow arrow denotes the blocking effect. (c) Schematic of the transport process of hydrated Zn^2+^, SO_4_^2−^, and free water through the angstrom‐scale confined Ti_0.87_O_2_ layer. (d) 2D Ti_0.87_O_2_ nanosheets with atomic Ti vacancies enable size‐selective exclusion of water and SO_4_^2−^, while allowing the passage of desolvated zinc ions. (e) 2D Ti_0.87_O_2_ nanosheets with angstrom‐scale free interlayer spacing enable size‐selective exclusion of water and SO_4_^2−^, while allowing the passage of desolvated zinc ions.

To date, significant efforts have been dedicated to tackling interfacial water parasitic reactions and dendrite growth, involving substrate morphology design, functional separator design, electrolyte configuration regulation, electrolyte additives, and artificial interfacial layer fabrication [8, 9]. Among these strategies, artificial interfacial layers hold greater potential for stabilizing zinc anodes owing to their ability to isolate the anode from the electrolyte, along with their practical applicability and material diversity [10]. Various organic and inorganic materials have been used as interfacial layers, including nano–CaCO_3_ [11], SiO_2_ [12], monolayer porous vermiculites (MPVMTs) [13], montmorillonite (MMT) [14], metal‐organic frameworks (MOFs) [10, 15], covalent organic frameworks (COFs) [16, 17], MXene [18], BN [19], and MoS_2_ [20], etc. These artificial interlayers have improved the zinc anode performance to some extent. However, considering the intrinsic sizes of hydrated zinc ions (∼8.6 Å), water molecules (∼4.0 Å), SO_4_^2−^ (∼5.9 Å), and Zn^2+^ (∼1.5 Å), they still face significant limitations in precisely regulating the migration of desolvated Zn^2+^, anions, hydrated zinc ions, and water molecules at the electrolyte‐anode interface (Figure 1a), thereby limiting their protective efficacy for zinc anodes [21].

Typically, for particle‐based materials, such as MOFs, COFs and inorganic particles, coatings prepared by slurry mixing often fail to form a fully dense and continuous interface, leaving interparticle gaps that may allow SO_4_^2−^ anions and water molecules to bypass the intrinsic pores and reach the Zn anode surface [22, 23]. For 2D materials, such as graphene oxide (GO), MXene, molybdenum disulfide (MoS_2_) and graphitic carbon nitride (g–C_3_N_4_), their effective pore structures are highly dependent on synthesis and post‐treatment conditions, and many reported pores remain in the nanoscale range (>10 Å) [24]. In addition, the interlayer channels of 2D materials are often enlarged by surface functional groups (e.g., ─OH, ─COOH, ─NH_2_, and ─SH), intercalated ions, and hydration‐induced swelling. In particular, oxygen–containing functional groups can form strong hydrogen bonds with water molecules, leading to enlarged hydrated interlayer spacing, typically above the angstrom‐scale range, and thus weakening the suppression of water penetration [25, 26]. Moreover, for MOF and COF monolayers, the ion/molecule channels are generally determined by organic linkers and framework packing, which often results in micro– or nanoscale channels (Table S1) [27, 28, 29, 30]. The precise sieving of hydrated Zn^2+^ ions, water molecules, SO_4_^2−^, and Zn^2+^ is fundamentally dependent on the material's internal pore size, which must be in the angstrom scale, smaller than water molecules (<4 Å) but larger than Zn^2+^ ions (>1.5 Å). This ensures selective and high‐permeability transport of Zn^2+^ ions while preventing the passage of water molecules and anions. It should be noted that the possible transport of H^+^/H_3_O^+^ cannot be completely excluded due to the slightly acidic nature of the ZnSO_4_ aqueous electrolyte. However, protons in aqueous solution generally exist not as isolated bare H^+^ ions, but as dynamically hydrated species, such as H_3_O^+^ or larger hydrated proton motifs, including Zundel‐type H_5_O_2_^+^ and Eigen‐type H_9_O_4_^+^ structures [31, 32]. These hydrated protonic motifs involve multiple water molecules and have an effective spatial extent exceeding the approximately 3.5 Å interlayer free spacing.

If a 2D material has a monolayer thickness and angstrom–scale pores and interlayer spacing (1.5–4 Å), it holds promise for precise sieving of hydrated Zn^2+^ ions, water molecules, SO_4_^2−^ anions, and desolvated Zn^2+^. Solvated Zn^2+^ ions undergo significant desolvation within angstrom‐scale confined ion channels, with desolvated Zn^2+^ ions transported homogeneously and rapidly through the channels. The solvation‐sheath water (released during Zn^2+^ desolvation), free water, and SO_4_^2−^ anions are effectively blocked on the exterior of the zinc anode, thereby suppressing side reactions involving water and SO_4_^2−^ at the zinc anode (Figure 1b).

Herein, we propose an angstrom‐scale confined Zn^2+^ ion accelerator for stabilizing aqueous Zn anodes through the selective regulation of hydrated Zn^2+^, desolvated Zn^2+^, H_2_O molecules, and SO_4_^2^^−^ anions (Figure 1c). Ti_0.87_O_2_ nanosheets with atomic in‐plane Ti vacancies (∼3.0 × 3.8 Å) and angstrom‐scale interlayer spacing (∼3.5 Å) are self‐assembled onto the Zn anodes. These distinctive in‐plane vacancies and interlayer‐confined channels synergistically promote Zn^2^^+^ enrichment, rapid dehydration of hydrated Zn^2^^+^, and shortened ion migration pathways, thereby functioning as a Zn^2^^+^ ion accelerator at the interface and enabling homogeneous Zn^2^^+^ transport with faster kinetics [33]. Meanwhile, they effectively block larger SO_4_^2−^ (∼5.9 Å) anions and free H_2_O molecules, resulting in a dendrite‐free zinc anode with suppressed parasitic reactions (Figure 1d,e). As a result, the Ti_0.87_O_2_@Zn anode demonstrates cycling lifespans of over 5000 and 4000 h at 1 and 5 mA cm^−2^, outperforming most artificial interfacial layer‐modified Zn anodes with comparable thickness reported in the literature. The as–prepared Ti_0.87_O_2_@Zn//NaV_3_O_8_·1.5H_2_O (NVO) and Ti_0.87_O_2_@Zn//VO_2_ full cells demonstrate capacity retentions of approximately 83.4% after 3000 cycles at 4 A g^−1^ and 86.2% after 2000 cycles at 3 A g^−1^, respectively. The Ah‐level Ti_0.87_O_2_@Zn//VO_2_ pouch cell, fabricated using a laminated assembly method, retains a high capacity of 85.4% of its initial capacity after 300 cycles at 3 A g^−1^. These results suggest that the Ti_0.87_O_2_ interfacial layer provides a useful design strategy for achieving highly selective and permeable ion diffusion, offering a new perspective for the next generation of aqueous zinc–ion batteries.

## 2 Results and Discussion

### 2.1 Synthesis and Characterization of Ti_0.87_O_2_@Zn Anodes

**FIGURE 2:**
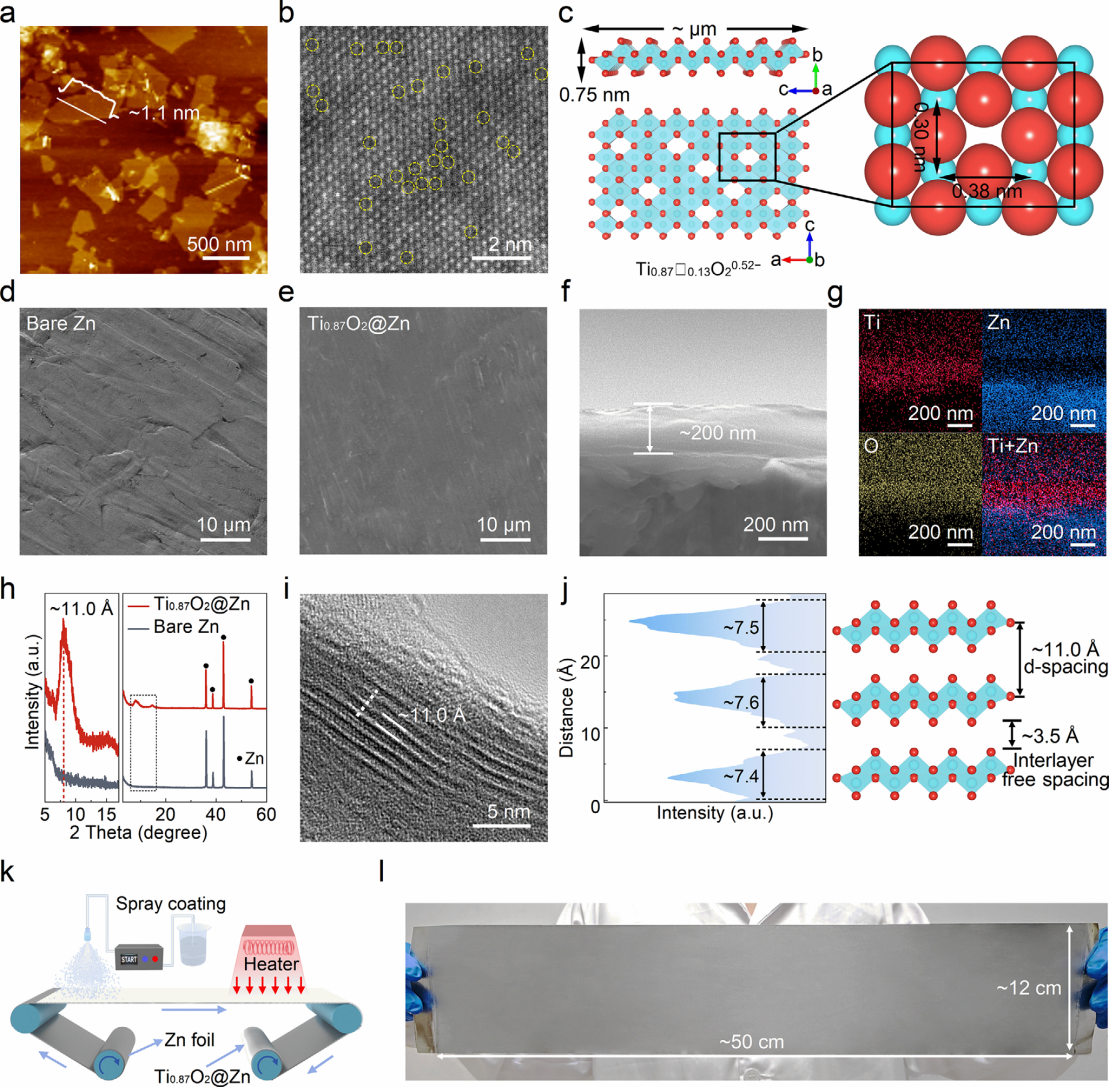
Synthesis and characterization of Ti_0.87_O_2_@Zn anodes. (a) AFM and (b) HAADF–STEM images of Ti_0.87_O_2_ nanosheets. The yellow circles in (b) indicate Ti vacancies. (c) Crystallographic structure of Ti_0.87_O_2_ nanosheets and the size of Ti vacancies shown with ionic radii. SEM images showing a top‐down view of (d) bare Zn and (e) Ti_0.87_O_2_@Zn. Cross‐sectional SEM images of (f) Ti_0.87_O_2_@Zn with a coating thickness of ∼200 nm and (g) the corresponding EDS mappings. (h) XRD patterns of bare Zn and Ti_0.87_O_2_@Zn, with the interlayer spacing of the nanosheets estimated to be ∼11.0 Å. (i) HRTEM images of Ti_0.87_O_2_ coating layer, with the coating layer ultrasonically fractured. (j) Atomic column intensity map from the HRTEM image of theTi_0.87_O_2_ coating layer, along with an illustration of the stacking arrangement and interlayer spacing of the coating. (k) Schematic illustration of industrially–applicable spray coating strategy. (l) Photograph of the fabricated Ti_0.87_O_2_@Zn with an area of 600 cm^2^, produced via a high–throughput manufacturing strategy.

The preparation process of titanium oxide nanosheets with varied vacancies (Ti_0.87_◻_0.13_O_2_^0.52−^ and Ti_0.91_◻_0.09_O_2_^0.36−^ and Ti_0.87_Li_0.13_O_2_^0.39−^) is comprehensively elucidated (Figure S1). In the first step, two kinds of layered lepidocrocite–type titanate crystals (K_0.8_Ti_1.73_Li_0.27_O_4_ and Cs_0.7_Ti_1.825_O_4_) were obtained through a high–temperature solid–state reaction (Figures S2, S3) [34]. Subsequently, an acid–exchange process was used to obtain layered titanate crystals intercalated with hydrated protons (H_0.7_Ti_1.825_O_4_·H_2_O and H_1.07_Ti_1.73_O_4_·H_2_O) (Figures S4, S5) [35, 36]. Finally, the two protonated samples were exfoliated in an aqueous solution of tetrabutylammonium hydroxide (TBAOH), forming a stable colloidal suspension of Ti_0.87_◻_0.13_O_2_^0.52−^ (Ti_0.87_O_2_) and Ti_0.91_◻_0.09_O_2_^0.36−^ (Ti_0.91_O_2_) nanosheets, both of which exhibited the Tyndall effect (Figure S6). In a control experiment, layered K_0.8_Ti_1.73_Li_0.27_O_4_ crystals were directly exfoliated in an N‐methyl‐D‐glucammonium (Meg^+^) solution to exchange interlayer K ions without leaching structural Li, resulting in a stable colloidal suspension of Ti_0.87_Li_0.13_O_2_^0.39−^ (Ti_0.87_Li_0.13_O_2_) nanosheets (Figure S6) [34]. Atomic force microscope (AFM) images confirmed that all as–obtained nanosheets of Ti_0.87_O_2_, Ti_0.91_O_2_, and Ti_0.87_Li_0.13_O_2_ showed monolayer characteristics with a similar thickness of approximately 1.1 nm (Figures 2a,b and S7). High‐angle annular dark‐field scanning transmission electron microscopy (HAADF‐STEM) images of the Ti_0.87_O_2_ and Ti_0.91_O_2_ nanosheets reveal the Ti vacancies and their distribution, with Ti_0.87_O_2_ nanosheets exhibiting a higher density of Ti vacancies compared to Ti_0.91_O_2_ (Figure 2b and S9). The corresponding line profiles exhibit variations in atomic column intensity, where the low‐intensity positions (indicated by arrows) are attributed to the formation of Ti vacancies (Figures S10, S11) [33, 37]. Electron paramagnetic resonance (EPR) spectroscopy shows that Ti_0.87_O_2_ exhibits stronger Ti and O vacancy signals compared to Ti_0.91_O_2_, while Ti_0.87_Li_0.13_O_2_ shows almost no vacancy signal, indicating that a higher concentration of Ti vacancies is present in Ti_0.87_O_2_ nanosheets (Figure S12) [38]. High‐resolution O 1s X‐ray photoelectron spectroscopy (XPS) spectra reveal an increase in binding energy in the order of R–TiO_2_, Ti_0.91_O_2_, and Ti_0.87_O_2_ nanosheets. This shift can be attributed to the O atoms adjacent to the Ti vacancies being closer to the stable eight‐electron configuration compared to the O atoms in pristine R–TiO_2_, further confirming the higher concentration of Ti vacancies in Ti_0.87_O_2_ nanosheets (Figure S13) [39]. Owing to the higher concentration of Ti vacancies, the Ti_0.87_O_2_ nanosheet suspension exhibits a more negative surface potential than the Ti_0.91_O_2_ and Ti_0.87_Li_0.13_O_2_ suspensions (Figure S14).

Subsequently, the suspensions of different nanosheets were sprayed onto Zn foil and exposed to ultraviolet (UV) light to obtain Ti_0.87_O_2_@Zn, Ti_0.91_O_2_@Zn, and Ti_0.87_Li_0.13_O_2_@Zn anodes. Compared to the rough surface of bare Zn (Figure 2d), the Ti_0.87_O_2_@Zn shows a smooth and uniform surface decorated by Ti_0.87_O_2_ nanosheets (Figure 2e). Cross–sectional scanning electron microscopy (SEM) images (Figure 2f) and corresponding energy‐dispersive X‐ray spectroscopy (EDS) mapping further verify the presence of the nanosheet modification layer (Figure 2g). The purpose of exposing Ti_0.87_O_2_@Zn to UV light is to photocatalytically decompose the TBA^+^ ions (Figure S15) [40]. As a result, the interlayer spacing of the Ti_0.87_O_2_ layers decreases significantly from ∼18.4 to ∼11.0 Å (Figure S16). Compared to the bare Zn anode, the additional diffraction peak with a repeated distance of ∼11.0 Å originates from the nanosheets being self–assembled onto the surfaces of Zn anodes (Figure 2h). Similar results can also be observed in Ti_0.91_O_2_@Zn and Ti_0.87_Li_0.13_O_2_@Zn (Figure S17). Further, the cross‐sectional high‐resolution transmission electron microscopy (HRTEM) image and the selected area electron diffraction (SAED) pattern of the ultrasonically fractured Ti_0.87_O_2_ layer reveal an interlayer spacing of ∼11.0 Å (Figures 2i and S18) [35]. The corresponding grayscale profile of the Ti_0.87_O_2_ coating layer confirms that the average monolayer thickness of the nanosheet is ∼0.75 nm, which is generally consistent with the crystallographic thickness of Ti_0.87_O_2_ nanosheets reported in previous literature (Figure 2j) [40, 41]. Thus, based on the results of HRTEM and XRD, the interlayer free spacing of the Ti_0.87_O_2_ coating layer is determined to be approximately 3.5 Å (Figure 2j) [40, 41].

By adjusting the concentration of the nanosheet suspension during the spraying process, coatings with varying thicknesses of nanosheet modifications were obtained (ranging from 50 to 800 nm) (Figure S19). A linear relationship between the nanosheet mass loading and the modification thickness of nanosheets on the Zn anode was observed (Figure S20). For comparison, the Ti_0.91_O_2_ and Ti_0.87_Li_0.13_O_2_ nanosheets were coated onto the Zn surfaces using a similar spray‐coating approach (Figures S21, S22). Besides, the coated Ti_0.87_O_2_ layers firmly adhere to the surfaces of Zn foils, maintaining intimate contact even during rolling, bending, and unfolding processes (Figure S23). Additionally, using an industrially scalable spraying technique (Figure 2k), the Ti_0.87_O_2_@Zn anode can be easily scaled up to 600 cm^2^, demonstrating considerable potential for practical applications (Figure 2l).

### 2.2 Ionic Transport Kinetics Within the Interfacial Layer

**FIGURE 3:**
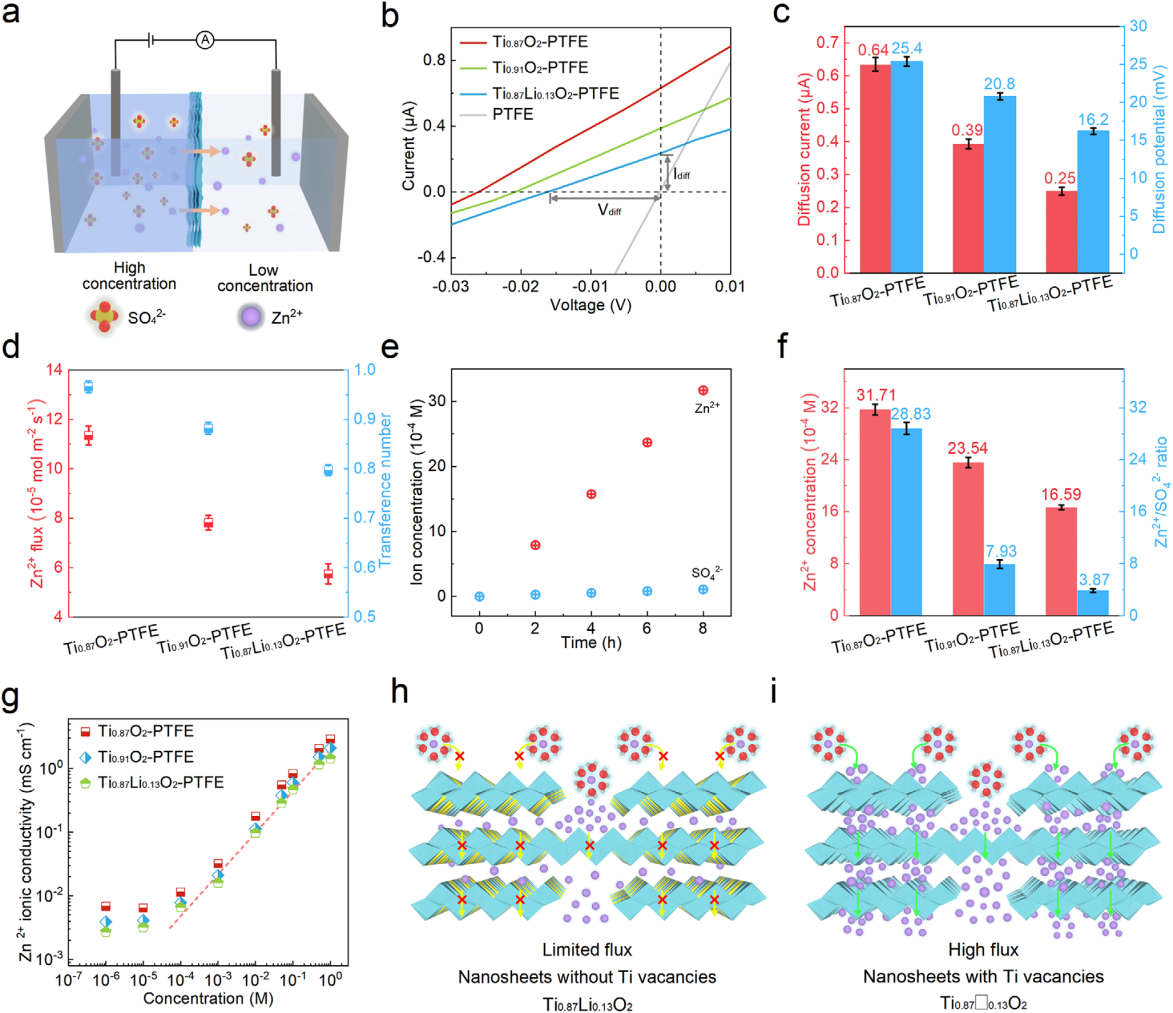
Zn^2+^ transport kinetics of Ti_0.87_O_2_ layer. (a) Schematic illustration of the testing setup for the permselective transport of Zn^2+^/SO_4_^2−^ across Ti_0.87_O_2_–PTFE, Ti_0.91_O_2_–PTFE, and Ti_0.87_Li_0.13_O_2_–PTFE membranes. (b) *I*–*V* curves of Ti_0.87_O_2_–PTFE, Ti_0.91_O_2_–PTFE, and Ti_0.87_Li_0.13_O_2_–PTFE measured under a transmembrane salinity gradient (0.5 M vs. 0.01 M ZnSO_4_ aqueous solution). (c) *I_diff_* and *V_diff_* values of Ti_0.87_O_2_–PTFE, Ti_0.91_O_2_–PTFE, and Ti_0.87_Li_0.13_O_2_–PTFE measured with 0.5 M and 0.01 M ZnSO_4_ aqueous solutions. (d) Calculated Zn^2+^ flux and transference numbers of Ti_0.87_O_2_–PTFE, Ti_0.91_O_2_–PTFE, and Ti_0.87_Li_0.13_O_2_–PTFE membrane. (e) Zn^2+^ and SO_4_^2−^ concentrations in the side of deionized water with Ti_0.87_O_2_–PTFE membrane during an 8 h test. (f) Zn^2+^ and SO_4_^2−^ concentrations on the deionized water side after 8 h of permeation through Ti_0.87_O_2_–PTFE, Ti_0.91_O_2_–PTFE, and Ti_0.87_Li_0.13_O_2_–PTFE membranes. (g) Zn^2+^ ionic conductivity measurement of different membrane recorded in ZnSO_4_ aqueous solutions at varying concentrations. Comparison of the schematics for Zn^2+^ transport in (h) Ti_0.87_Li_0.13_O_2_ and (i) Ti_0.87_£_0.13_O_2_ nanosheets. The presence of Ti vacancies provides higher flux for Zn^2+^.

Fast interfacial Zn^2+^ transport kinetics on the zinc anode are crucial for the design of artificial interfacial layers in high‐rate AZMBs [10, 12]. To investigate the transport kinetics of Zn^2+^ within the nanosheet interfacial layers, a diffusion experiment was conducted using nanosheet‐coated polytetrafluoroethylene (PTFE) as a barrier within an H–type electrochemical cell (Figure 3a). The experimental setup involves introducing 0.01 M ZnSO_4_ electrolyte on the left side and 0.5 M ZnSO_4_ on the right side of the H‐type cell. Driven by concentration differences, Zn^2+^ and SO_4_^2−^ selectively pass through the nanosheet membrane and complete the circuit with electrons in the external wiring, thereby generating an osmosis–driven transmembrane voltage and current [42]. By applying an opposing current or voltage in the external circuit, the osmosis–driven transmembrane voltage and current can be fully compensated, allowing their intrinsic values to be quantified [43, 44]. It should be noted that the possible transport of H^+^/H_3_O^+^ cannot be completely excluded because of the weakly acidic nature of the ZnSO_4_ aqueous electrolyte. Nevertheless, under the actual condition of 0.5 M ZnSO_4_, Zn^2+^ remains the predominant cationic charge carrier. To isolate the influence of Ti vacancies, all samples were subjected to UV irradiation for 2 h to remove interlayer TBA^+^, ensuring that the interlayer spacing of the nanosheets was controlled to be identical across different samples. Compared to Ti_0.91_O_2_–PTFE (∼20.8 mV and ∼−0.392 µA) and Ti_0.87_Li_0.13_O_2_–PTFE (∼16.2 mV and ∼−0.248 µA), the as‐assembled H‐type cell with Ti_0.87_O_2_–PTFE as barrier exhibits the highest open–circuit voltage (*V_oc_*) (∼25.4 mV) and short‐circuit current (*I_sc_*) (∼–0.634 µA) (Figure 3b,c). A high *V_oc_* indicates a greater selective transport of Zn^2+^ through the nanosheet membrane, while a high *I_sc_* reflects the enhanced permeability and high flux of Zn^2+^ ions [45]. As a reference, the PTFE substrate exhibits negligible selective ion transport for Zn^2+^ and SO_4_^2−^ ions (with a transference number of ∼0.5), providing a negligible influence on the permeation current and voltage (Figure 3b). Based on the obtained *V_oc_* and *I_sc_*, the Zn^2+^ flux and transference numbers are further quantified. The Ti_0.87_O_2_‐PTFE exhibited the highest Zn^2+^ flux (11.35 × 10^−5^ mol m^−2^ s^−1^) and selectivity (96.6 %) compared to Ti_0.91_O_2_‐PTFE (7.82 × 10^−5^ mol m^−2^ s^−1^ and 88.2%) and Ti_0.87_Li_0.13_O_2_‐PTFE (5.75 × 10^−5^ mol m^−2^ s^−1^ and 79.7%) (Figure 3d), demonstrating the accelerated and highly selective Zn^2+^ ion transport at the Ti_0.87_O_2_ interface layer. Such an acceleration effect originates from the negatively charged Ti vacancies and angstrom‐scale channels, which enrich Zn^2^^+^ near the interface, facilitate confined desolvation, and provide shortened pathways for fast Zn^2^^+^ migration [33, 40, 46].

Furthermore, the same H–type electrochemical cell without applied voltage was used to investigate the transport behavior of Zn^2+^ and SO_4_^2−^ across different nanosheet layers, with the feed side filled with 0.5 M ZnSO_4_ solution and the permeate side containing deionized water. Inductively Coupled Plasma Mass Spectrometry (ICP‐MS) was employed to quantify the concentrations of Zn^2+^ and SO_4_^2−^ on the permeate side. After 8 h of ion‐free diffusion, the Zn^2+^ concentration (31.71 × 10^−4^ mol L^−1^) on the deionized water side of Ti_0.87_O_2_‐PTFE was significantly higher than that of Ti_0.91_O_2_‐PTFE (23.54 × 10^−4^ mol L^−1^) and Ti_0.87_Li_0.13_O_2_–PTFE (16.59 × 10^−4^ mol L^−1^). Meanwhile, the Ti_0.87_O_2_–PTFE exhibits the highest Zn^2+^/SO_4_^2−^ selectivity ratio (28.83), far exceeding that of Ti_0.91_O_2_‐PTFE (7.93) and Ti_0.87_Li_0.13_O_2_‐PTFE (3.87) (Figures 3e,f and S24). The spontaneous Zn^2^^+^ permeation observed in the bias–free osmosis experiment indicates that Zn^2^^+^ transport across the Ti_0.87_O_2_ layer is not solely driven by an externally applied electric field. Instead, the concentration‐gradient‐driven Zn^2^^+^ migration suggests that the Ti_0.87_O_2_ layer provides favorable pathways for selective Zn^2^^+^ transport. Considering the angstrom–scale interlayer spacing and in‐plane vacancy channels, hydrated Zn^2^^+^ may undergo partial solvation‐sphere reconstruction when passing through the confined Ti_0.87_O_2_ layer. Therefore, the Ti_0.87_O_2_ layer functions beyond simple ion sieving by facilitating Zn^2^^+^ desolvation, thereby reducing transport resistance and promoting rapid Zn^2^^+^ migration across the interfacial layer. In addition, the pH on the deionized water side is monitored to assess the transmembrane migration of protons or acidic species. With bare PTFE, the receiving‐side pH decreased from approximately 6.8 to 4.19 after 8 h. By contrast, the pH remained at approximately 6.08 when the Ti_0.87_O_2_‐PTFE membrane was used, close to that of deionized water exposed to air under the same conditions (∼6.21) (Figure S25). The decrease in the pH of deionized water may be caused by the dissolution of CO_2_ in the air [47]. These results indicate that the Ti_0.87_O_2_ nanosheet layer substantially suppresses the crossover of protonic or acidifying species.

Besides, the ionic conductivity of the nanosheet membranes was evaluated across ZnSO_4_ solutions of varying concentrations, with current‐voltage (*I–V*) measurements recorded for Ti_0.87_O_2_‐PTFE, Ti_0.91_O_2_‐PTFE and Ti_0.87_Li_0.13_O_2_‐PTFE in symmetric electrolytes from 10^−6^ to 1 M. The *I–V* curves at different concentrations exhibit linear ohmic behavior, and the conductivity of the nanosheets was calculated from the slope (Figure S26). As shown in Figure 3g, Ti_0.87_O_2_–PTFE exhibited higher Zn^2+^ ionic conductivity than Ti_0.91_O_2_–PTFE and Ti_0.87_Li_0.13_O_2_–PTFE across all concentrations, indicating a faster Zn^2+^ transport in the Ti_0.87_O_2_ layer. The relationship between ionic conductivity and concentration can be divided into two regions. Taking Ti_0.87_O_2_‐PTFE as an example, when the ZnSO_4_ concentration exceeds 10^−3^ M, its conductivity decreases linearly with the concentration of ZnSO_4_, exhibiting properties similar to bulk ZnSO_4_. When ZnSO_4_ concentration is below 10^−3^ M, Ti_0.87_O_2_‐PTFE exhibits unique Zn^2+^ transport behavior, with conductivity remaining constant at 2 × 10^−^^2^ mS cm^−1^. This indicates surface‐charge‐governed Zn^2+^ transport behavior, suggesting active ion transport by the nanosheets at low concentrations [48, 49]. Given that the structural variation among the nanosheets is primarily defined by the number of in‐plane vacancies, it can be concluded that the vacancies are the primary factor dominating the Zn^2^^+^ transport flux and kinetics, alongside a secondary contribution to the Zn^2+^/SO_4_^2−^ selectivity (Figure 3h,i).

### 2.3 Investigation of Interfacial Desolvation and Water Transport Mechanism

**FIGURE 4:**
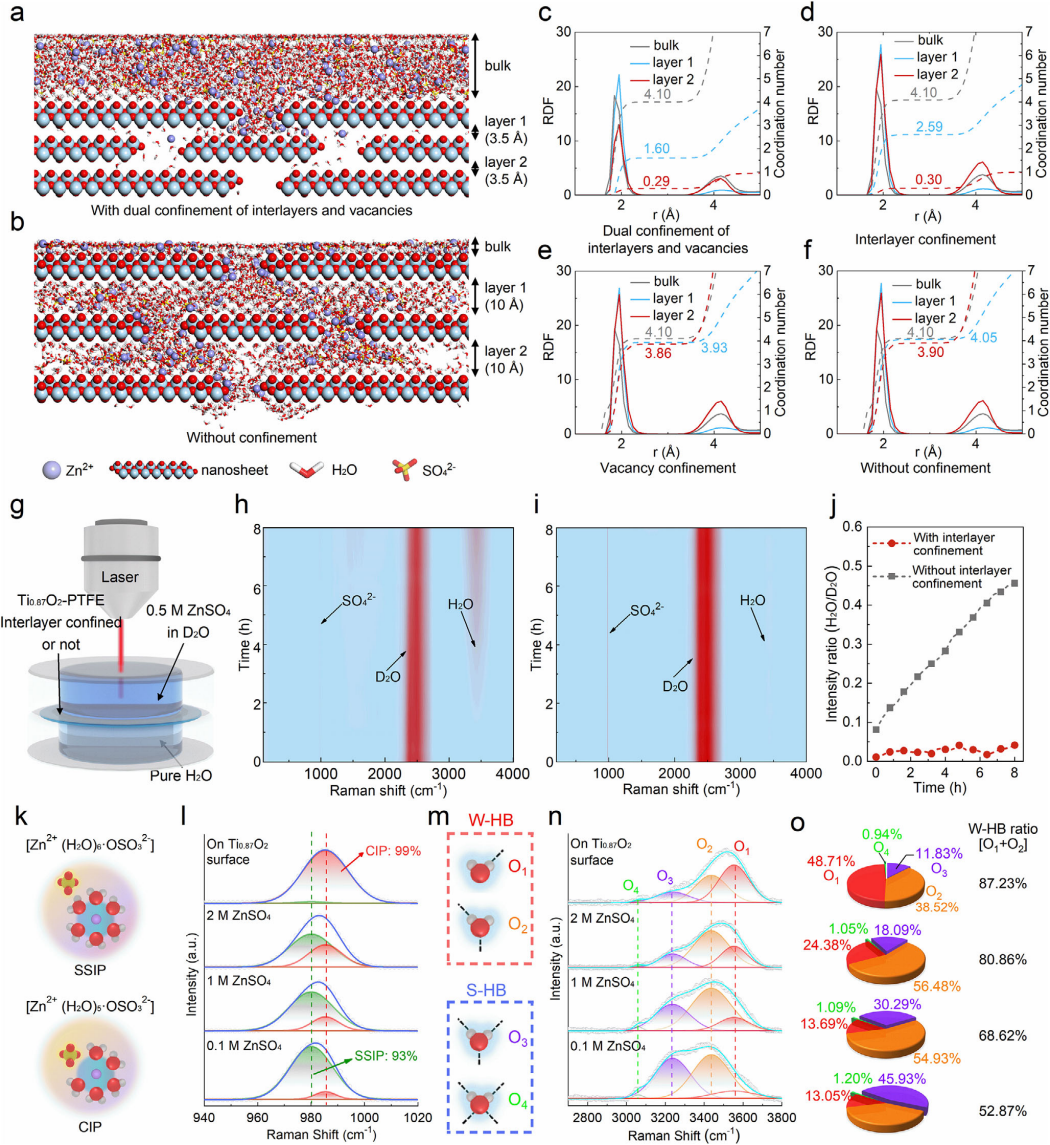
Investigation of the interfacial desolvation and water transport mechanism. Snapshots of the MD simulations taken at 100 ps for 2 M ZnSO_4_ electrolyte diffusion across the stacked nanosheets with (a) dual confinement of interlayers and vacancies and (b) without confinement. The corresponding RDF plots of (c) dual confinement of interlayers and vacancies, (d) single interlayer confinement, (e) single vacancy confinement, and (f) without confinement in the selected areas of the bulk electrolyte, the first interlayer (lay 1), and the second interlayer (lay 2) of the nanosheet. Schematic illustration of the in situ Raman setup used for testing water transmembrane transport. (g) In situ Raman spectra of the solution in the upper chamber using Ti_0.87_O_2_–PTFE barriers (h) without and (i) with interlayer confinement during an 8–h static water permeation experiment. (j) The corresponding Raman intensity ratio of H_2_O to D_2_O. (k) Schematic illustration of CIP and SSIP of SO_4_^2−^. (l) Raman spectra of the ν–SO_4_^2−^ band of ZnSO_4_ aqueous solution with concentrations ranging from 0.1 to 2 M and on the Ti_0.87_O_2_ surface. (m) Schematic illustration of weakly hydrogen‐bonded water species, W–HB (O_1_ and O_2_), and strongly hydrogen‐bonded water species, S─HB (O_3_ and O_4_). (n) Raman spectra of the O─H stretch vibration and (o) corresponding proportions of O_1_, O_2_, O_3_, and O_4_ in ZnSO_4_ aqueous solution with different concentrations and on the Ti_0.87_O_2_ surface.

Molecular dynamics (MD) simulations were employed to investigate the coupled ion‐transport and solvation‐sphere regulation behavior of hydrated Zn^2+^ during its migration through angstrom‐scale confined ion channels. To decouple the individual effects of vacancies and interlayer spacing on ion/molecule transport and hydrated Zn^2+^ desolvation, four models (including dual confinement of interlayers and vacancies, single confinement of interlayers, single confinement of vacancies, and no confinement) were constructed to simulate the desolvation behavior of hydrated zinc ions within stacked nanosheets (Figure S27). The presence or absence of Ti vacancies and different interlayer spacings (3.5 and 10.0 Å) represent in–plane vacancies and interlayer confinement, respectively. In the process of concentration–driven diffusion, [Zn(H_2_O)_6_]^2+^, H_2_O molecules, and SO_4_^2−^ anions spontaneously migrated across the nanosheet layers, during which snapshots were taken (Figures 4a,b and S28 and S29). For the model with dual confinement of interlayers and vacancies, at 100 ps, the hydration number of Zn^2+^ exhibits a significant decrease from the bulk electrolyte to the nanosheet layers. Specifically, it decreases from 4.10 in the bulk electrolyte to 1.60 in the first interlayer of the nanosheet, and further to 0.29 in the second layer (Figure 4c). This is attributed to the dual angstrom‐scale confined ion channels effectively hindering the entry of water molecules and SO_4_^2−^, leading to the low coordination of Zn^2+^, indicating the superior desolvation effect of the dual angstrom‐scale confined nanosheets. For comparison, under single interlayer confinement, the hydration number of Zn^2+^ decreases to 2.59 in the first interlayer and 0.30 in the second (Figure 4d). Under the single vacancy confinement, it decreases to 3.93 in the first interlayer and 3.86 in the second (Figure 4e). In the absence of confinement, the hydration number of Zn^2+^ decreases to 4.05 in the first interlayer and 3.90 in the second (Figure 4f). These results demonstrate that the angstrom‐scale confined interlayer spacing primarily governs the desolvation of hydrated Zn^2+^, whereas in‐plane vacancies play a subordinate role in this specific process. Besides, the temperature‐dependent electrochemical impedance spectroscopy (EIS) was employed to investigate the Zn^2+^ desolvation facilitation effect of the Ti_0.87_O_2_ interfacial layer during the Zn^2+^ plating process (Figure S30). The activation energy (*E_a_*) for Zn^2+^ desolvation can be quantitatively fitted using the Arrhenius equation [16, 50, 51]. According to the fitting results, the Ti_0.87_O_2_@Zn symmetric cells exhibit the lowest *E_a_* (21.1 kJ mol^−1^) compared with Ti_0.91_O_2_@Zn (28.7 kJ mol^−1^), Ti_0.87_Li_0.13_O_2_@Zn (38.3 kJ mol^−1^) and bare Zn (54.1 kJ mol^−1^) (Figure S31), confirming that the Ti_0.87_O_2_ interfacial layer reduces the kinetic barrier for Zn^2^^+^ desolvation and accelerates interfacial Zn^2^^+^ transport.

For a more intuitive demonstration of the inhibition of water transport by the nanosheet interlayer confinement, a self‐made vertical H‐type cell was constructed. The TBA‐intercalated Ti_0.87_O_2_ interlayer was coated onto a PTFE substrate as the barrier, and the interlayer spacing was precisely regulated from ∼11 Å (without confinement) to 3.4 Å (interlayer confined) by controlling the removal of interlayer TBA via UV irradiation (Figure 4g), following a method adapted from the preparation of Ti_0.87_O_2_@Zn. The lower and upper chambers of the H‐type cell were filled with pure H_2_O and 0.5 M ZnSO_4_ D_2_O solution, respectively. Under the influence of osmotic pressure, Zn^2+^ and SO_4_^2−^ spontaneously transport downwards, while H_2_O tends to move upwards. The Raman vibration differences between D_2_O and H_2_O were utilized to monitor the penetration behavior of D_2_O and H_2_O across the Ti_0.87_O_2_ interfacial layer (Figure S32). As shown in Figure 4h,i, in the H‐type cell with Ti_0.87_O_2_–PTFE without interlayer confinement, H_2_O easily permeates between the two chambers during an 8‐h standing test. In contrast, in the H‐type cell utilizing Ti_0.87_O_2_‐PTFE with interlayer confinement, H_2_O encounters difficulty in permeating through the Ti_0.87_O_2_‐PTFE layer to reach the chamber containing 0.5 M ZnSO_4_ (Figure 4j), demonstrating the superior ability of the Ti_0.87_O_2_ interfacial layer to resist water transport. As a control, in the H‐type cell utilizing only PTFE as the barrier, a greater amount of H_2_O permeates between the two chambers during the 8–h standing test, eventually reaching a steady state (Figure S33). Efficient long‐range Grotthuss proton transport requires a sufficiently connected and dynamically reorganizable hydrogen‐bonded water network [32], the inhibited water transport through the Ti_0.87_O_2_ layer also suggests restricted proton transport.

To further investigate the impact of the Ti_0.87_O_2_ interfacial layer on the transport of hydrated Zn^2+^, water molecule, and SO_4_^2−^, detailed Raman analysis was conducted on the surface of the Ti_0.87_O_2_ layer after cycling at 1 mA cm^−2^ for 1 h in a 2 M ZnSO_4_ electrolyte. For aqueous ZnSO_4_ electrolyte, a gradual red shift of the ν–SO_4_^2−^ bond is observed as the concentration of the electrolyte increases from 0.1 M to 2 M. The ν–SO_4_^2−^ bond, centered at 980–985 cm^−1^, can be attributed to two main species: solvent‐shared ion pairs (SSIP, [Zn(H_2_O)_6_·SO_4_^2−^] located at about 980 cm^−1^) and contact ion pairs (CIP, [Zn^2+^ (H_2_O)_5_·SO_4_^2−^] located at about 985 cm^−1^) (Figure 4k) [10, 12]. The results indicate that the coordination of Zn^2+^ with solvated water is inhibited in more concentrated solutions, leading to the desolvation of the typical [Zn(H_2_O)_6_]^2+^ complex. In comparison, the surface of Ti_0.87_O_2_ layer exhibits a higher concentration of SO_4_^2−^ within the solvation shell of Zn^2+^, with a CIP ratio of approximately 99%, which may be caused by the negatively charged Ti_0.87_O_2_ nanosheets repelling SO_4_^2−^ and forming a SO_4_^2−^–rich layer (Figure 4l). Additionally, the stretching vibration of the O─H bond was used to characterize the hydrogen‐bonding state of water on the surface of Ti_0.87_O_2_ nanosheets. The O─H stretching vibration can be deconvoluted into four components, denoted as O_1_, O_2_, O_3_, and O_4_ (Figures 4m and S34). Among these, O_3_ and O_4_ represent fully hydrogen‐bonded water molecules, while O_1_ and O_2_ represent partially hydrogen–bonded water molecules (Figure 4m) [10]. As shown in Figure 4n,o, the W‐HB ratio increases from 52.87% in 0.1 M ZnSO_4_ to 80.86% in 2 M ZnSO_4_, indicating that concentrated ZnSO_4_ electrolyte weakens the hydrogen‐bonding network of water. Notably, the Ti_0.87_O_2_ surface exhibits an even higher W‐HB ratio of 87.23%, accompanied by a decreased S‐HB ratio of 12.77%. These results indicate that water molecules near the Ti_0.87_O_2_ surface are involved in a weaker and less extended hydrogen–bonding network. This interfacial restructuring is likely associated with the negatively charged Ti_0.87_O_2_ nanosheets, which promote Zn^2+^ accumulation and alter the local ion‐pairing and solvation environment at the nanosheet/electrolyte interface [12, 40]. The resulting ion‐enriched interfacial region perturbs the original water–water hydrogen–bonding network and increases the proportion of W–HB water [52]. It should be noted that the Raman results primarily reflect changes in the collective hydrogen‐bonding environment of water rather than directly demonstrating a weakening of the Zn^2+^–O coordination bonds. Nevertheless, water molecules that are less extensively involved in the cooperative hydrogen‐bonding network can undergo more facile structural reorganization during interfacial ion transport [53, 54]. Therefore, the increased W–HB fraction may facilitate reconstruction of the Zn^2+^ solvation shell and promote partial desolvation as hydrated Zn^2+^ enters the angstrom‐scale confined channels. Since Zn^2+^ desolvation is a critical interfacial step preceding Zn deposition, such regulation of the interfacial water structure is consistent with the reduced desolvation barrier and enhanced charge‐transfer kinetics observed for Ti_0.87_O_2_@Zn.

### 2.4 Highly Reversible Zinc Anodes with Dendrite Suppression, Side Reaction Suppression, and Fast Kinetics

The zinc deposition and side reaction behavior of the zinc anode during cycling were further investigated to illustrate the stabilizing effect of the nanosheet interfacial layer on the zinc anode. Density Functional Theory (DFT) calculations were conducted to investigate the mechanism of the nanosheets with Ti vacancies to regulate Zn^2+^ deposition. To simplify the model, Lepidocrocite TiO_2_ monolayers with different vacancies were constructed, where the double‐vacancy and single‐vacancy structure corresponds to Ti_0.87_O_2_ and Ti_0.91_O_2_ respectively. For comparison, vacancy‐free Lepidocrocite TiO_2_ and bare Zn were also modeled to provide a comprehensive reference. The optimized models indicate that Zn atoms preferentially adsorb at the sites in the TiO_2_ monolayer where Ti atoms are missing. In contrast, when no Ti atoms are missing, the Lepidocrocite TiO_2_ model exhibits a slight repulsion toward Zn, which can be attributed to the negative electric field generated by the absence of Ti (Figure S35). Adsorption energy calculations further reveal that Ti_0.87_O_2_ exhibits the strongest adsorption (−8.611 eV), followed by Ti_0.91_O_2_ nanosheets (−7.945 eV) and bare Zn (−0.591 eV), while lepidocrocite TiO_2_ shows a slight repulsion (0.241 eV) (Figure S36). These results suggest that Zn^2^^+^ preferentially adsorbs at Ti vacancy sites, indicating that the negatively charged vacancies act as Zn^2^^+^‐attracting centers to enrich Zn^2^^+^ and homogenize the interfacial ion flux, which is a key component of the Zn^2^^+^ ion–acceleration effect. Furthermore, electrostatic potential (ESP) calculations for Ti_0.87_O_2_, Ti_0.91_O_2_, and lepidocrocite TiO_2_ also reveal that the O atoms near the Ti vacancies exhibit a higher charge density, indicating that these O atoms around the vacancy sites are more likely to attract Zn^2+^ ions (Figure S37). Figure S38 illustrates the transfer profile of single Zn^2+^ passing through a lepidocrocite TiO_2_ monolayer with or without Ti vacancy. For lepidocrocite TiO_2_ monolayer, the potential‐energy barrier reaches 51.1 eV, while it is only 0.58 eV for TiO_2_ monolayer with Ti vacancy (Figure S39). This indicates that Ti vacancies provide low‐barrier in‐plane transport pathways for Zn^2^^+^ migration, further supporting the role of Ti_0.87_O_2_ as a Zn^2^^+^ ion accelerator rather than merely a passive ion filter.

The Ti_0.87_O_2_ layer enriched with Ti vacancies facilitates the rapid diffusion of Zn^2+^, resulting in a higher Zn^2+^ diffusion coefficient (*D_Zn_^2+^* ═ 9.62 × 10^−6^ for Ti_0.87_O_2_@Zn, compared to *D_Zn_^2+^* ═ 4.60 × 10^−6^ for Ti_0.87_O_2_@Zn, *D_Zn_^2+^* ═ 1.17 × 10^−6^ for Ti_0.87_Li_0.13_O_2_@Zn and *D_Zn_^2+^* ═ 5.56 × 10^−7^ for bare Zn) (Figure S40, S41). Benefiting from the accelerated Zn^2+^ transport kinetics, the concentration polarization of symmetrical cells of Ti_0.87_O_2_@Zn decreases from 18.7 mV to 5.5 mV, while both ohmic and electrochemical polarization were reduced from 51.3 mV in bare Zn to 19.9 mV, respectively. For comparison, Ti_0.91_O_2_@Zn (22.8 and 6.3 mV) and Ti_0.87_Li_0.13_O_2_@Zn (32.8 and 11.4 mV) exhibit relatively lower effects in balancing polarization, which can be attributed to the slower Zn^2+^ migration kinetics caused by the reduced vacancies among the nanosheets (Figure S42).

**FIGURE 5:**
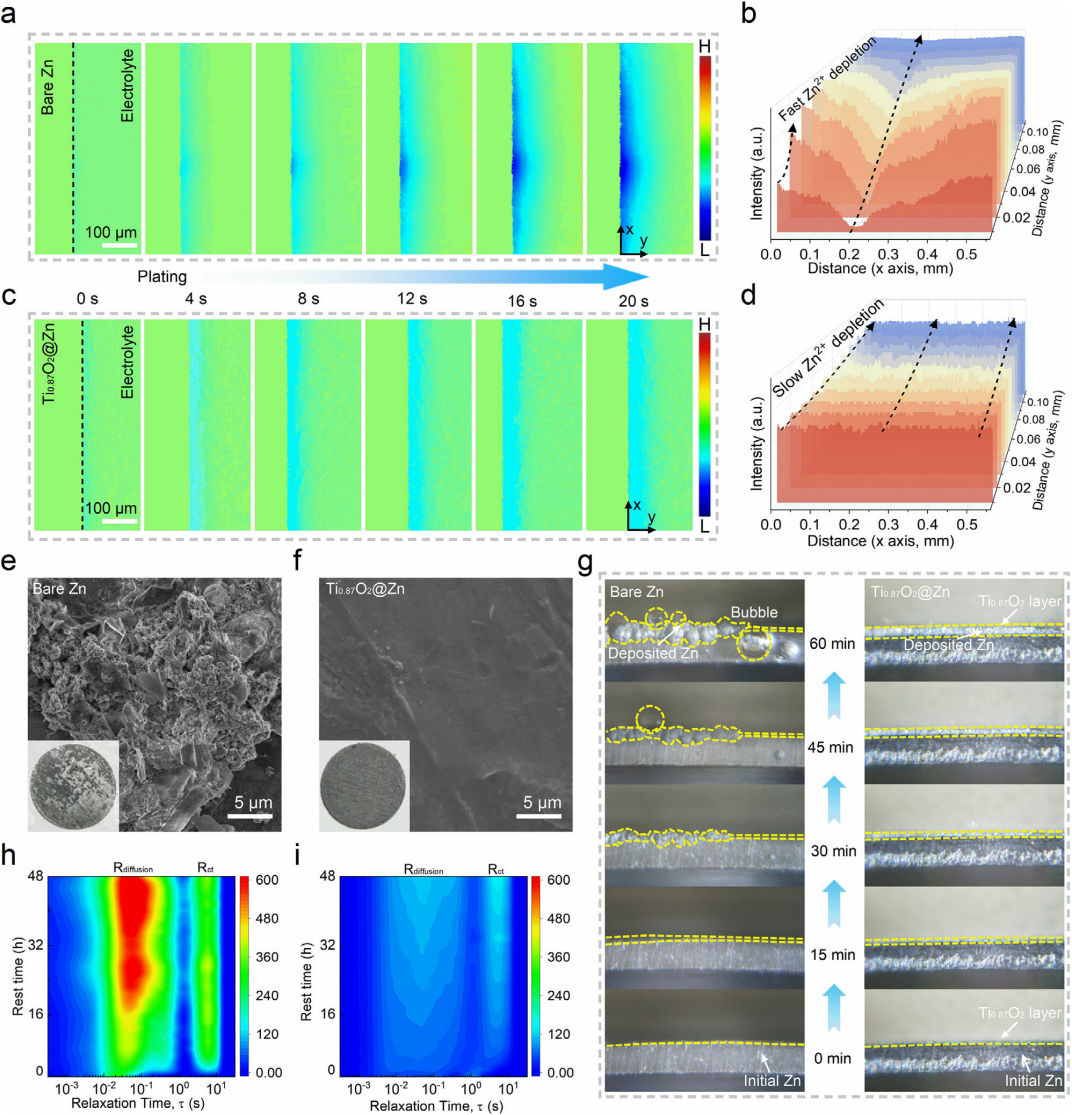
Zinc anodes with dendrite suppression, side reaction suppression, and fast kinetics. In situ EDH projection of (a) bare Zn and (c) Ti_0.87_O_2_@Zn anode at different deposition times, along with the (b,d) corresponding 3D area charts of Zn^2+^ concentration along the normal direction of anode at 15 s. SEM images of bare Zn (e) and Ti_0.87_O_2_@Zn (f) after 20 cycles at 1 mA cm^−2^ and 1 mAh cm^−2^. The inset shows digital photographs of the anodes after 20 cycles. (g) In situ optical microscopy images of bare Zn and Ti_0.87_O_2_@Zn anode at 1 mA cm^−2^. DRT analysis of EIS evolution in resting (h) bare Zn and (i) Ti_0.87_O_2_@Zn symmetric cells.

In situ electrochemical digital holography (EDH) was employed to monitor the ion concentration evolution during the initial Zn^2+^ reduction at the electrolyte‐anode interface by measuring optical path length distribution [12, 55]. As shown in Figure 5a,c, after 20 s of electrochemical reduction, a noticeable local color change is observed on the bare Zn surface, suggesting rapid and localized depletion of Zn^2+^ at the interface. This diffusion‐limited local concentration gradient should trigger the imminent growth of Zn dendrites [12]. In contrast, the Ti_0.87_O_2_ layer on the Zn anode surface reorganizes the Zn^2+^ flux, promoting uniform and rapid Zn^2+^ transport at the interface, providing direct evidence for its Zn^2+^ ion‐accelerating effect during electrodeposition. The 3D area charts from EDH show the corresponding concentration distribution after 20 s of electrodeposition (Figure 5b,d). The surface of the Ti_0.87_O_2_@Zn anode exhibits a uniform concentration distribution with a small concentration gradient, whereas the bare Zn surface shows pronounced local ion depletion and a heterogeneous concentration profile. This directly demonstrates that the Ti_0.87_O_2_ layer effectively reorganizes the surface Zn^2+^ concentration distribution, facilitating the deposition kinetics of the zinc anode.

The diffusion behavior of Zn^2+^ ions was further investigated through chronoamperometry (CA). Unlike the rampant 2D diffusion observed on bare zinc anode, the Ti_0.87_O_2_@Zn, Ti_0.91_O_2_@Zn, and Ti_0.87_Li_0.13_O_2_@Zn maintain a stable diffusion current, indicating a strong guiding effect of Ti_0.87_O_2_ interfacial layer to Zn^2+^ and stable ion transport (Figure S43). SEM and digital images after cycling were employed to monitor the zinc deposition behavior. As revealed by SEM after 20 plating/stripping cycles at 1 mA cm^−2^, the bare Zn anode displayed extensive dendritic structures caused by non‐uniform Zn deposition (Figure 5e). Meanwhile, the surfaces of Ti_0.87_O_2_@Zn and Ti_0.91_O_2_@Zn remained smooth and continuously coated with their respective interfacial layers, suggesting uniform Zn growth and minimal interfacial degradation (Figures 5f and S44). By comparison, Ti_0.87_Li_0.13_O_2_@Zn exhibited a rougher surface after cycling, likely due to the loss of vacancy‐induced ion‐regulation capability (Figure S45). Digital images of the zinc anode after cycling provide a more direct visualization (Figures 5e,f, and S44). In detail, the surface of the bare zinc anode exhibits numerous protrusions, with deposited zinc accumulating at these sites, leaving large areas of the zinc anode without deposition. In contrast, after cycling, the surfaces of Ti_0.87_O_2_@Zn, Ti_0.91_O_2_@Zn, Ti_0.87_Li_0.13_O_2_@Zn are uniformly covered with a thin layer of deposited Zn, demonstrating effective dendrite suppression. The morphology of Zn beneath the Ti_0.87_O_2_ interfacial layer was further examined. At the edge of the Zn substrate, the Ti_0.87_O_2_ layer was manually peeled off, exposing the underlying Zn surface. The exposed Zn exhibited a well‐aligned and densely packed structure, highlighting the strong guiding effect of the Ti_0.87_O_2_ interphase on Zn^2+^ ion flux (Figure S45). To monitor the real‐time Zn deposition process, in situ optical microscopy was employed on the bare Zn and Ti_0.87_O_2_ anodes during continuous electroplating at a current density of 1 mA cm^−^^2^ for 60 min (Figure S46). As revealed by in situ optical microscopy, the bare Zn anode exhibited pronounced dendrite growth and bubble formation over a 60 min period, indicating uncontrolled Zn nucleation and concurrent hydrogen evolution reactions (Figure 5g). In contrast, Ti_0.87_O_2_@Zn and Ti_0.91_O_2_@Zn displayed uniform and compact layer growth, whereas the vacancy‐blocked Ti_0.87_Li_0.13_O_2_@Zn showed relatively disordered deposition morphology (Figure S47). To further clarify the preferred crystallographic orientation of deposited Zn, ex situ XRD analysis was performed on the initial Zn foil, bare Zn after plating, and Ti_0.87_O_2_@Zn after plating. As shown in Figure S48, the relative peak–area fractions of Zn(002), Zn(100), and Zn(101) are 43.7%, 11.0%, and 45.3% for the initial Zn foil; 35.6%, 11.7%, and 52.7% for bare Zn after plating; and 26.3%, 13.6%, and 60.1% for Ti_0.87_O_2_@Zn after plating, respectively. These semi‐quantitative results indicate that the Ti_0.87_O_2_ interfacial layer promotes preferential Zn(101)‐textured deposition rather than a dominant Zn(002) orientation. Previous studies have shown that Zn(101)‐textured growth can also support dense, directional, and reversible Zn deposition, indicating that uniform deposition is not exclusively associated with the Zn(002) plane [17, 56, 57]. Therefore, the uniform Zn deposition on Ti_0.87_O_2_@Zn is attributed to the combined effects of regulated crystallographic orientation, homogenized Zn^2^^+^ flux, facilitated interfacial desolvation and transport, and spatially uniform nucleation and growth.

Furthermore, the effect of Ti_0.87_O_2_ interfacial layers in corrosion resistance was further investigated. During a 48‐h resting test, symmetric cells with bare Zn anode showed pronounced peaks of diffusion resistance (*R_diffusion_*) and charge transfer resistance (*R_ct_*) in the optimized distribution of relaxation times (DRT), indicating severe water‐related side reactions and corrosion (Figures 5h and S49). In contrast, symmetric cells with Ti_0.87_O_2_@Zn anodes exhibited a relatively stable interface impedance change, indicating robust water resistance and enhanced stability of the zinc anode (Figures 5i and S50). Tafel analysis further confirmed the superior corrosion resistance of Ti_0.87_O_2_@Zn, which exhibited a higher corrosion potential and a significantly lower corrosion current density (1.99 mA cm^−2^) compared to Ti_0.91_O_2_@Zn (2.56 mA cm^−2^), Ti_0.87_Li_0.13_O_2_@Zn (3.16 mA cm^−2^) and bare Zn (4.63 mA cm^−2^) (Figure S51). Similarly, the hydrogen evolution reaction (HER) on the Ti_0.87_O_2_@Zn electrode is significantly suppressed, attributable to the effective isolation of active water molecules from the Zn surface by the Ti_0.87_O_2_ interfacial layer (Figure S52). XRD analysis was further conducted to assess the interfacial layer's ability to stabilize the Zn anode. The bare Zn electrode exhibited distinct by‐product peaks, indicative of severe water‐induced side reactions. In contrast, Ti_0.87_O_2_@Zn showed negligible by‐product formation, confirming the layer's effectiveness in mitigating electrolyte‐induced corrosion during cycling (Figure S53). The structural integrity of the Ti_0.87_O_2_ interfacial layer was retained after electrochemical cycling. Both the well‐defined lamellar morphology (Figure S54) and the Ti vacancy structure (Figure S55) remained stable after 50 cycles at 1 mA cm^−2^ with a capacity of 1 mAh cm^−2^. To further provide chemical–state evidence, post‐cycling XPS characterization was newly performed. The Ti 2p and O 1s spectra of Ti_0.87_O_2_@Zn after cycling show well‐preserved characteristic signals of Ti_0.87_O_2_ without obvious peak shifts or the formation of new chemical states (Figure S56), confirming the excellent chemical stability of the Ti_0.87_O_2_ interfacial layer. These results collectively demonstrate the robust structural and chemical stability of Ti_0.87_O_2_ during long‐term electrochemical cycling.

### 2.5 Performance Evaluation of Highly Reversible Zn Anode

**FIGURE 6:**
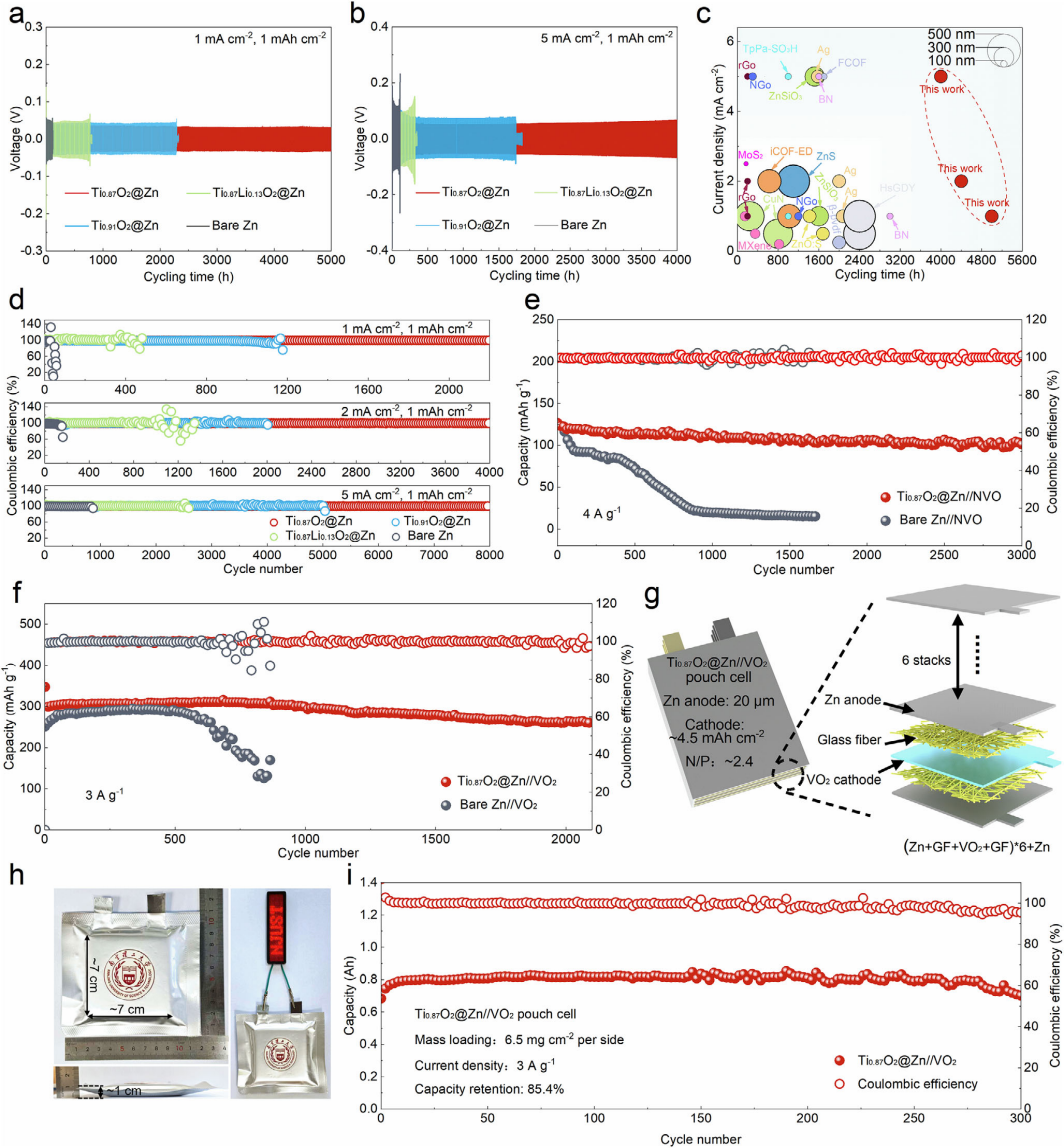
Electrochemical performance of Ti_0.87_O_2_@Zn anodes. Cycling performance of Ti_0.87_O_2_@Zn, Ti_0.91_O_2_@Zn, Ti_0.87_Li_0.13_O_2_@Zn, and bare Zn symmetric cells at (a) 1 mA cm^−2^ and (b) 5 mA cm^−2^ with a capacity of 1 mAh cm^−2^. (c) Comparison of the cycling lifespan of symmetric cells at 1, 2, and 5 mA cm^−2^ with previous reported interfacial layers–modified Zn anodes with comparable thickness [13, 17, 18, 19, 20, 61, 62, 63, 64, 65, 66, 67, 68, 69, 70]. (d) Coulombic efficiencies of Ti_0.87_O_2_@Zn//Cu, Ti_0.91_O_2_@Zn//Cu, and bare Zn//Cu cells at 1, 2, and 5 mA cm^−2^ with an area capacity of 1 mAh cm^−2^. (e) Cycling performance of Ti_0.87_O_2_@Zn//NVO and bare Zn//NVO cells at 4 A g^−1^. (f) Cycling performance of Ti_0.87_O_2_@Zn//VO_2_ and bare Zn// VO_2_ cells at 3 A g^−1^. (g) The schematic images of Ti_0.87_O_2_@Zn//VO_2_ pouch cell. (h) Digital photograph showing the size and thickness of the Ti_0.87_O_2_@Zn//VO_2_ pouch cell, along with a photo of the pouch cell continuously powering a light panel. (i) Cycling performance of the Ti_0.87_O_2_@Zn// VO_2_ pouch cell at 3 A g^−1^.

Rapid Zn^2+^ transport kinetics, dense Zn deposition and efficient interface desolvation enabled by nanosheet interfacial layers are expected to significantly enhance the electrochemical performance of Zn anodes. Initially, the effect of the thickness of Ti_0.87_O_2_ layer on the cycling stability of Zn//Zn symmetric cells was investigated. Thicker coatings may block the ion transport between Zn foil and the electrolyte, resulting in a high overpotential, while extremely thin coatings exhibit lower cycling stability due to weak mechanical strength [18, 58]. As a result, a coating thickness of ∼200 nm offers the best balance of cycling stability and relatively low overpotential (Figure S57), thereby establishing it as the standardized thickness for subsequent stability evaluations. As shown in Figure 6a, the Ti_0.87_O_2_@Zn and Ti_0.91_O_2_@Zn anodes maintained stable plating/stripping voltage profiles for over 5000 h and 2200 h at 1 mA cm^−2^, respectively. In comparison, the Ti_0.87_Li_0.13_O_2_@Zn anodes showed stability for over ∼740 h, while the bare Zn anodes failed after ∼86 h due to dendrite‐induced short‐circuiting [59]. Increasing the current density will lead to an uneven electric field at the anode, resulting in reduced yet unfavorable nucleation [60]. At a current density of 2 mA cm^−2^, the cell with the bare Zn failed after ∼79 h, while the Ti_0.87_O_2_@Zn, Ti_0.91_O_2_@Zn, and Ti_0.87_Li_0.13_O_2_@Zn symmetric cells operated for over 4400, 2480, and 580 h, respectively (Figure S58). Furthermore, even under a high current density of 5 mA cm^−2^, the Ti_0.87_O_2_@Zn symmetric cell exhibited a long lifespan of over 4000 h with superior voltage stability, compared to 1709 h for Ti_0.91_O_2_@Zn, ∼350 h for Ti_0.87_Li_0.13_O_2_@Zn. Whereas the bare Zn anode showed an irregularly fluctuating voltage profile and polarization after only ∼130 h (Figure 6b). The rate performance of Ti_0.87_O_2_@Zn and Ti_0.91_O_2_@Zn as Zn anodes were also investigated at current densities ranging from 0.2 to 10 mA cm^−2^ (Figure S59). The overpotentials of Ti_0.87_O_2_@Zn, Ti_0.91_O_2_@Zn, Ti_0.87_Li_0.13_O_2_@Zn and bare Zn were compared at different current densities (Figure S60). As a result, the Ti_0.87_O_2_@Zn symmetric cells exhibited lower polarization across different current densities compared to Ti_0.91_O_2_@Zn and Ti_0.87_Li_0.13_O_2_@Zn, especially at high current densities, which may be attributed to the reduced vacancies that limit Zn^2+^ transport within the interfacial layers. Notably, when the performance of symmetric cells is compared at different current densities (1, 2, and 5 mA cm^−2^), our protective layer provides a level of stability for the zinc anode that significantly outperforms most artificial interfacial layer‐modified Zn anodes with comparable thickness reported in the literature, indicating its great potential for practical applications (Figure 6c and Table S2).

To further evaluate the practical applicability of Ti_0.87_O_2_@Zn under more realistic working conditions, intermittent galvanostatic charge/discharge tests were conducted at 1 mA cm^−^^2^ and 1 mAh cm^−^^2^. As shown in Figure S61, the Ti_0.87_O_2_@Zn symmetric cell maintained a stable voltage profile and low polarization for 1020 h under repeated intermittent plating/stripping processes, whereas the bare Zn cell exhibited unstable voltage fluctuation and failed within a much shorter cycling time. This result indicates that the Ti_0.87_O_2_@Zn can effectively withstand repeated electrochemical operation and resting‐induced interfacial corrosion. In addition, a depth‐of‐discharge test was performed using thin Zn foil with a thickness of 20 µm under a high areal capacity of 10 mAh cm^−^^2^, corresponding to a Zn utilization of approximately 85.5% DOD. At 5 mA cm^−^^2^ and 10 mAh cm^−^^2^, the Ti_0.87_O_2_@Zn symmetric cell delivered a stable cycling performance for 400 h with relatively low voltage hysteresis. In contrast, the bare Zn cell showed more obvious polarization fluctuation and inferior cycling stability (Figure S62). These results demonstrate that the Ti_0.87_O_2_ interfacial layer can effectively stabilize Zn plating/stripping even under high Zn utilization and limited Zn‐reservoir conditions. The plating/stripping reversibility of Ti_0.87_O_2_@Zn, Ti_0.91_O_2_@Zn, and Ti_0.87_Li_0.13_O_2_@Zn anodes was also evaluated in asymmetric Zn//Cu asymmetric cells. The Zn//Cu asymmetric cell with Ti_0.87_O_2_@Zn as the anode demonstrated stable cycling for over 2200 cycles and maintained a stable voltage profile for more than 4400 h at a current density of 1 mA cm^−2^, indicating excellent stability and Zn plating/stripping reversibility (Figure 6d). In comparison, the Ti_0.91_O_2_@Zn//Cu and Ti_0.87_Li_0.13_O_2_@Zn//Cu cells maintained stable cycling for over 1150 and 380 cycles, respectively. The bare Zn cell remained relatively stable in voltage only during the first 1–30 cycles, after which it failed due to short‐circuiting caused by dendrite formation. Additionally, the voltage profile of the Ti_0.87_O_2_@Zn//Cu asymmetric cell exhibited lower polarization voltage (61 mV) compared to Ti_0.91_O_2_@Zn//Cu (65 mV), Ti_0.87_Li_0.13_O_2_@Zn//Cu (74 mV) and bare Zn//Cu (107 mV) at 1 mA cm^−2^, further confirming that the Ti_0.87_O_2_@Zn anode exhibited better deposition kinetics (Figure S63). Similarly, the plating/stripping stability of Ti_0.87_O_2_@Zn and Ti_0.91_O_2_@Zn in asymmetric cells under higher current conditions (2 and 5 mA cm^−2^) was investigated. The Ti_0.87_O_2_@Zn anode also exhibited higher charge/discharge efficiency and stability (Figure 6d).

### 2.6 Full Cell Performance

The practical applicability of the Ti_0.87_O_2_@Zn anode was demonstrated using two cathodes by evaluating the electrochemical performance of Ti_0.87_O_2_@Zn//NVO and Ti_0.87_O_2_@Zn//VO_2_ full cells. The as–prepared NaV_3_O_8_·1.5H_2_O (NVO) and VO_2_ exhibit morphologies and XRD patterns consistent with those reported in the literature (Figure S64) [71, 72]. Cyclic voltammetry (CV) curves of the Ti_0.87_O_2_@Zn//NVO and Ti_0.87_O_2_@Zn//VO_2_ full cell both exhibit a lower oxidation peak and a higher reduction peak, indicating that the Ti_0.87_O_2_ coating reduces electrochemical polarization and enhances the electrochemical activity of the full cell (Figure S65). Further, the long‐term cycling performance of the Ti_0.87_O_2_@Zn//NVO and Ti_0.87_O_2_@Zn//VO_2_ full cells were tested. After 3000 cycles, the Ti_0.87_O_2_@Zn//NVO full cell retains over 83% of its initial capacity at 4 A g^−1^ (Figure 6e), whereas the Ti_0.87_O_2_@Zn//VO_2_ full cell maintains ∼86% capacity retention at 3 A g^−1^ (Figure 6f). The capacity stability of Ti_0.87_O_2_@Zn//NVO and Ti_0.87_O_2_@Zn//VO_2_ full cells exceed that of most materials reported in the literature, highlighting its superior electrochemical performance (Table S3). For Ti_0.87_O_2_@Zn//VO_2_ and bare Zn//VO_2_ full cells, the surface morphology of the Ti_0.87_O_2_@Zn and bare zinc anodes were examined after cycling. After 50 cycles at a current density of 4 A g^−1^, the Ti_0.87_O_2_@Zn anode maintained a relatively smooth and uniform surface, whereas the bare Zn anode exhibited uneven Zn deposition and pronounced dendrite growth under identical conditions (Figure S66). In addition, the self‐discharge behavior of Ti_0.87_O_2_@Zn//VO_2_ and bare Zn//VO_2_ full cells was evaluated. The Ti_0.87_O_2_@Zn‐based full cell retained 98.5% of its capacity after resting, in contrast to only 89.0% for the bare Zn counterpart (Figure S67). These results collectively indicate that the nanosheet‐based interfacial layer effectively stabilizes the Zn anode by suppressing parasitic reactions and Zn corrosion, thereby mitigating self‐discharge and dendrite formation. To further validate the device–relevant practicality of the Ti_0.87_O_2_@Zn anode, an Ah‐level Ti_0.87_O_2_@Zn//VO_2_ pouch cell was successfully fabricated using a laminated configuration (Figure 6g,h). The pouch cell delivers a high areal capacity of approximately 167.3 mA h cm^−2^ and maintains 85.4% of its initial capacity after 300 cycles, demonstrating remarkable cycling stability under practical conditions (Figure 6i).

## 3 Conclusion

In summary, we employed an angstrom‐scale confined ion accelerator strategy to achieve highly stable aqueous Zn anodes by selectively and precisely regulating the transport of hydrated Zn^2+^, Zn^2+^ cations, H_2_O molecules, and SO_4_^2−^ anions. The angstrom‐scale confined vacancies and interlayer structure of the Ti_0.87_O_2_ nanosheet layer effectively restrict the permeation of SO_4_^2−^ anions and H_2_O molecules, enabling efficient dehydration of hydrated zinc ions and facilitating the uniform passage of Zn^2+^ cations with enhanced transport kinetics. This precise control over ion transport within the Ti_0.87_O_2_ interfacial layer results in fast Zn^2+^ transport, effective interfacial dehydration, and superior suppression of dendrites and side reactions. As a result, the Ti_0.87_O_2_@Zn anode demonstrates an extended cycling lifespan, exceeding 5000 h at 1 mA cm^−2^ and 4000 h at 5 mA cm^−2^. The Ti_0.87_O_2_@Zn//NaV_3_O_8_·1.5H_2_O and Ti_0.87_O_2_@Zn//VO_2_ full cells retain approximately 83% and 86% of their initial capacity after 3000 and 2000 cycles at 4 A g^−1^ and 3 A g^−1^, respectively. Additionally, the Ah–level Ti_0.87_O_2_@Zn//VO_2_ pouch cell, fabricated using the laminated method, retains 85.4% of its initial capacity after 300 cycles at 3 A g^−1^. The angstrom‐scale confined ion accelerator strategy provides a novel approach for achieving highly selective and permeable ion diffusion, offering a promising path forward for the development of next‐generation aqueous zinc‐based and other metal‐ion batteries.

## 4 Experimental Methods

### 4.1 Preparation of Ti_0.87_O_2_, Ti_0.91_O_2_, and Ti_0.87_Li_0.13_O_2_ Nanosheets

For Ti_0.87_O_2_ nanosheets, the colloidal suspension was obtained via exfoliation of layered titanate precursors. Typically, K_2_CO_3_, Li_2_CO_3_, and TiO_2_ were mixed and ground homogeneously at a molar ratio of 0.8:0.27:3.46, the resulting mixture was annealed at 1273 K for 20 h to synthesize the layered titanate precursor (K_0.8_Ti_1.73_Li_0.27_O_4_). Subsequently, the sample was reacted in 1 M HCl solution for 3 days to form a proton‐intercalated intermediate (H_1.07_Ti_1.73_O_4_·H_2_O). The HCl solution was replaced with a fresh solution daily. The solid product was obtained by filtration, followed by washing with deionized water and air‐drying. Subsequently, the proton‐intercalated titanate (H_1.07_Ti_1.73_O_4_·H_2_O) was shaken in a tetrabutylammonium hydroxide (TBA^+^) aqueous solution. The molar ratio of the concentration of TBA^+^ to the number of exchangeable protons in titanate was 1:1. After 7 days, a stable suspension of Ti_0.87_O_2_ nanosheets was obtained. For Ti_0.91_O_2_ nanosheets, CS_2_CO_3_, and TiO_2_ were mixed and ground homogeneously at a molar ratio of 0.7:3.65 to form the Cs_0.7_Ti_1.825_O_4_ layered titanate precursor. The proton‐intercalated titanate H_0.7_Ti_1.825_O_4_·H_2_O was obtained by acid exchange with 1 M HCl for 24 h, with the acid solution being replaced three times. The remaining steps followed the synthesis procedure for Ti_0.87_O_2_ nanosheets. For Ti_0.87_Li_0.13_O_2_ nanosheets, the as‐prepared K_0.8_Ti_1.73_Li_0.27_O_4_ was dispersed and stirred in 40 mL of 1 M meglumine (Meg) solution with a pH value of 9, adjusted using HCl solution (37% aqueous solution). The meglumine solution was replaced every 12 h, following centrifugation at 10,000 rpm for 5 min, repeated three times. The resulting Meg‐titanate was washed twice with deionized water and ethanol to remove excess meglumine and then dried in air. Subsequently, 100 mg of Meg–titanate powder was mixed with 25 mL of deionized water and shaken in an overhead shaker for 1 week.

### 4.2 Preparation of Ti_0.87_O_2_@Zn, Ti_0.91_O_2_@Zn, and Ti_0.87_Li_0.13_O_2_@Zn Anode

The Ti_0.87_O_2_@Zn anode was prepared by spraying a stable Ti_0.87_O_2_ nanosheet suspension onto a zinc foil. For the fabrication of Ti_0.87_O_2_@Zn, a suspension of Ti_0.87_O_2_ nanosheets (0.25 mL, 4 mg mL^−1^) and an aqueous solution of PTFE binder were mixed at a mass ratio of 9:1 in a water/ethanol mixture (v/v ═ 1:3). The solution was then ultrasonicated for 10 min to ensure uniform mixing. Subsequently, the solution was uniformly sprayed onto polished zinc foil (30 cm^2^) using a spray gun, while the substrate was heated on an 80°C hot plate to facilitate rapid solvent evaporation. The obtained Ti_0.87_O_2_@Zn anode was dried and then exposed to ultraviolet (UV) light for 2 h before being assembled into electrochemical cells. The estimated nanosheet loading on the zinc foil was approximately 0.03 mg cm^−2^, which could be adjusted by varying the mass concentration during the spraying process. The same procedure was followed for synthesizing Ti_0.91_O_2_@Zn and Ti_0.87_Li_0.13_O_2_@Zn.

### 4.3 Preparation of Ti_0.87_O_2_–PTFE, Ti_0.91_O_2_–PTFE, Ti_0.87_Li_0.13_O_2_–PTFE

Suspensions of Ti_0.87_O_2_, Ti_0.91_O_2_, and Ti_0.87_Li_0.13_O_2_ nanosheets were spray‐coated onto commercial hydrophilic PTFE membranes (PTFE membranes were purchased from [www.gooddelv.com]). The areal mass loading of Ti_0.87_O_2_ was controlled at ∼0.03 mg cm^−2^. The resulting Ti_0.87_O_2_–coated PTFE films were air–dried and irradiated under UV light for 2 h to obtain the final Ti_0.87_O_2_–PTFE samples. Ti_0.91_O_2_–PTFE and Ti_0.87_Li_0.13_O_2_–PTFE were prepared using the same procedure.

### 4.4 Characterization

AFM was conducted using a Dimension 3100 SPM instrument to analyze the topography of nanosheets. STEM images were acquired with a Titan Cube Themis G2 300 transmission electron microscope. The micro‐textures and morphologies of the samples were examined by SEM (Hitachi S4800) and TEM (JEOL JEM–ARM200F). XRD patterns were recorded on a Bruker D8 Advance diffractometer operated at 40 kV and 40 mA, using Cu–Kα radiation (λ ═ 1.5418 Å). Water contact angles were measured at room temperature with an OCA25 contact angle goniometer (DataPhysics OCA 35, Germany). XPS was performed on a PHI Quantera II system. Zeta potentials of nanosheet suspensions were determined using a Malvern ZS90 analyzer. Raman spectra were collected using a Leica Microsystems inVia Raman microscope equipped with Labspec software. EPR spectra were obtained from a Bruker EMXPLUS spectrometer. The growth process and morphology of zinc dendrites were monitored in real‐time using a custom‐built symmetric cell tester under an in situ optical microscope.

### 4.5 H–Type Electrochemical Cell

I–V curves were measured using a Keithley 2634B source meter (Keithley Instruments). A dual–chamber H–type electrochemical cell was employed to evaluate Zn^2+^ permeation performance, with an effective testing area of approximately 0.03 cm^2^. 0.5 M and 0.01 M ZnSO_4_ solutions were placed in the two chambers, with Ti_0.87_O_2_–PTFE, Ti_0.91_O_2_–PTFE, Ti_0.87_Li_0.13_O_2_–PTFE, and PTFE serving as barriers. The side containing the vacuum‐filtered nanosheets was oriented toward the 0.5 M ZnSO_4_ solution. The transmembrane electrical potential and current were recorded using a salt bridge Ag/AgCl reference electrode (AME–RO302).

For the calculation of the Zn^2+^ transference number and Zn^2+^ flux, Zn^2+^ diffuses from high‐to low‐concentration regions under a concentration gradient. The cation transference number (t_+_) is given by the following equation:

(1) $$t_{+}=-\frac{1}{2}\left(\frac{V_{diff}}{\frac{RT}{zF}In\left(\frac{r_{c_{H}}c_{H}}{r_{c_{L}}c_{L}}\right)}+1\right)$$

where *V_diff_* denotes the diffusion potential, *R* is the universal gas constant, *T* represents the absolute temperature, *z* is the charge number, F is the Faraday constant, *r* refers the ion activity coefficient, and *c* is the ion concentration.

The Zn^2+^ flux can be calculated by the following equation:

(2) $$J_{Zn^{2+}}=-\frac{I}{zF}\left(\frac{t_{+}}{t^{+}-t^{-}}\right)$$

where *JZn^2+^* denotes the zinc ion flux (mol m^−2^ s^−1^), *I* represents the current (A m^−2^), *z* is the charge number of the zinc ion, and F is the Faraday constant.

### 4.6 Symmetric and Asymmetric Cell Measurements

The stripping and deposition stability of symmetric and asymmetric cells was evaluated using a LANDdt (Shenzhen) battery test system. CR2025 coin‐type batteries were assembled with 50 µm thick zinc foil, a Whatman GF/D glass fiber separator, and 2.0 M ZnSO_4_ electrolyte. The assembled batteries were allowed to rest for 2 h before electrochemical testing to ensure complete penetration at the electrode interface. The CE of the asymmetric cell was assessed using a Cu//Zn configuration, with a cutoff voltage set at 0.6 V. An optical microscope (AW33T–4k, Hangzhou Aoweisi Technology Co., Ltd.) and a custom‐made transparent symmetric cell were employed for in situ observations. CA characterization was performed on a symmetric cell at a constant voltage of −150 mV using the VMP3 electrochemical workstation. GITT profiles were obtained at 1 mA cm^−2^ per cycle, with a one‐min charging step followed by a one‐min rest period, using a LANDdt battery test system. EIS was conducted on a Bio‐Logic electrochemical workstation (CHI760E), covering a frequency range from 100 kHz to 10 mHz. The desolvation process of [Zn(H_2_O)_6_]^2+^, reflecting the water resistance of the coating layer, was analyzed via the desolvation activation energy (E_a_), determined using the Arrhenius equation.

(3) $$\frac{1}{R_{ct}}=Aexp\left(\frac{E_{a}}{RT}\right)$$

where *R_ct_* is the charge transfer resistance. *A* is the frequency factor, *R* is the gas constant, and *T* is the absolute temperature.

The distribution relaxation time (DRT) in the EIS data was calculated using the DRT toolbox developed by Professor Francesco Ciucci's research group in MATLAB R2019. DRT–TOOLS is available for free under the GNU license and can be accessed at the following website: https://sites.google.com/site/drttools/.

### 4.7 Theoretical Calculations

DFT calculations were conducted using the Vienna Ab‐initio Simulation Package (VASP). The electronic wave functions were expanded using a plane‐wave basis with a cutoff energy of 520 eV. The Brillouin zone (BZ) was sampled using a 3 × 1 × 3 Gamma Scheme k‐point mesh. All atomic positions were fully relaxed until the force was smaller than 0.01 eV/Å, and the energy tolerance was less than 1.0 × 10^−5^ eV per atom. A vacuum space of 15 Å was introduced to prevent unwanted interactions between atoms in adjacent cells. The adsorption energy (E_ad_) was calculated using the following equation:

(4) $$E_{ad}=E_{total}-E_{surface}-E_{Zn}$$

in which *E_total_*, *E_surface_*, and *E_Zn_* represent the total energy of the facet combined with the Zn, the energy of the clean surface, and the energy of the Zn, respectively.

### 4.8 Molecular Dynamics

MD simulations were performed using large‐scale atomic/molecular massively parallel simulator (LAMMPS) software. The lepidocrocite TiO_2_ and Ti_0.87_O_2_ nanosheet structures were constructed based on a previous study [73]. The atomic charges were determined via first–principles calculations [74]. The net charge of titanium atoms in the TiO_2_ nanosheet was 1.32e, while the oxygen atoms carried charges of −0.71e or −0.60e. In the Ti_0.87_O_2_ nanosheet, the net charge of titanium atoms ranged from 1.31e to 1.33e, while that of oxygen atoms varied between −0.69e and −0.59e. 5000 water molecules, 180 SO_4_^2−^ ions, and 180 Zn^2+^ ions were contained in the boxes. The optimized potentials for liquid simulations (OPLS–AA) force field were applied to SO_4_^2−^ molecules, while the SPC/E force field was applied to water molecules [75]. Coulombic and van der Waals interactions were calculated using the 6–12 Lennard–Jones potential with a 1.2 nm cutoff distance. Simulations were performed in a 133.71 × 30.63 × 200.00 Å^3^ box, incorporating a 50 Å vacuum layer above the electrolyte and below the lowest nanosheet. Each system underwent energy minimization at 0 K, with energy and force tolerances set to obtain the ground‐state structure. The SETTLE algorithm was employed to maintain fixed bond lengths and angles in water molecules. Subsequently, the system was heated from 300 to 800 K in 5 ns and then cooled back to 300 K in 2 ns to expedite equilibrium. The total simulation time was 100 ns, using a 2–fs time step. Periodic boundary conditions were applied in all directions, and long–range electrostatic interactions were corrected via the particle‐particle particle‐mesh (PPPM) method.

## Author Contributions

X.P., P.X., and J.Z. designed the research. X.P., Z.L., K,L., C.L., and J.F. conducted the synthesis, characterizations, and electrochemical measurements. C.Y., Y.L., and N.S. performed the theoretical calculations. H.H., Z.H., W.Z., M.C., L.H., and Y.F. joined the discussion of data and gave useful suggestions. X.P., P.X., G.W., and J.Z. analyzed and discussed the experimental results and drafted the manuscript.

## Funding

J.Z. and P.X. acknowledge support from the National Natural Science Foundation of China (No. 52125202, 52472213, and U24A2065), the Natural Science Foundation of Jiangsu Province (BK20230035) and the Fundamental Research Funds for the Central Universities (No. 30925020208). G.W. acknowledges the support provided by the Australian Research Council (ARC) through the ARC Industry Laureate Fellowship (IL240100042) and the ARC Discover project (DP240102176 and DP230101579). L.H. acknowledge support from the National Natural Science Foundation of China (No. 52171203 and 52371214). C.Y. acknowledge support from the National Natural Science Foundation of China (No. 52473235), the National Key R&D Program of China (2022YFA1203400) and the Guangdong Provincial Key Laboratory of Computational Science and Material Design (2019B030301001). Computing resources were supported by the Center for Computational Science and Engineering at Southern University of Science and Technology.

## Conflicts of Interest

The authors declare no conflicts of interest.

## Supporting information




**Supporting File**: adma74289‐sup‐0001‐SuppMat.docx.

## Data Availability

All data are available within the Article and Supplementary Information or available from the first author and corresponding author on reasonable request.
