# Supplementary material for: Angstrom‐Scale Confined Ion Sieve and Accelerator for Efficient Aqueous Zinc Batteries
Source: Adv Mater. 2026 Jul 29;38(53):e74289. doi: 10.1002/adma.74289 (PMC13591656; doi:10.1002/adma.74289)
Supplement: Supplementary file 1 [file ADMA-38-e74289-s001.docx]

**Supplementary Information**

**Angstrom-Scale Confined Ion** **Sieve and Accelerator for Efficient Aqueous Zinc Batteries**

*Xing Peng, Caichao Ye, Yingqiang Li,* *Zhihang Liu, Kunxi Luan,* *Jinbo Fan, Honglan Huang, Chao Liu, Na Shen, Zhen Hou, Wenyao Zhang, Yongsheng Fu, Mingzhe Chen, Linfeng Hu, Pan Xiong**^*^, Guoxiu Wang^*^, Junwu Zhu^*^*

X. Peng, Y. Li, Z. Liu, K. Luan, J. Fan, H. Huang, Z. Hou, W. Zhang, Y. Fu, P. Xiong, J. Zhu.

Key Laboratory for Soft Chemistry and Functional Materials of Ministry of Education, School of Chemistry and Chemical Engineering, Nanjing University of Science and Technology, Nanjing, 210094, China

E-mail: pan.xiong@njust.edu.cn; zhujw@njust.edu.cn

C. Ye, N. Shen.

Academy for Advanced Interdisciplinary Studies & Department of Materials Science and Engineering, Guangdong Provincial Key Laboratory of Computational Science and Material Design, Southern University of Science and Technology, Shenzhen, 518055, China

C. Liu, G. Wang.

Centre for Clean Energy Technology, School of Mathematical and Physical Sciences, Faculty of Science, University of Technology Sydney, Sydney, New South Wales, 2007, Australia.

E-mail: Guoxiu.wang@uts.edu.au

M. Chen.

School of Energy and Power Engineering, Nanjing University of Science and Technology, Nanjing, 210094, China

L. Hu.

School of Materials Science and Engineering, Southeast University, Nanjing, 211189, China.

**Figure S1.** Schematic illustration of the synthesis of Ti_0.87_O_2_, Ti_0.91_O_2_, and Ti_0.87_Li_0.13_O_2_ nanosheets with an ion exchange-assisted exfoliation method.

**Figure S2.** SEM image of **(a)** Cs_0.7_Ti_1.825_O_4_ and **(b)** K_0.8_Ti_1.73_Li_0.27_O_4_.

**Figure S3.** XRD pattern of **(a)** Cs_0.7_Ti_1.825_O_4_ and **(b)** K_0.8_Ti_1.73_Li_0.27_O_4_.

**Figure S4.** SEM image of **(a)** H_0.7_Ti_1.825_O_4_·H_2_O and **(b)** H_1.07_Ti_1.73_O_4_·H_2_O.

**Figure S5.** XRD pattern of **(a)** H_0.7_Ti_1.825_O_4_·H_2_O and **(b)** H_1.07_Ti_1.73_O_4_·H_2_O.

**Figure S6.** Tyndall effect of Ti_0.87_O_2_, Ti_0.91_O_2_, and Ti_0.87_Li_0.13_O_2_ nanosheets suspensions.

**Figure S7.** AFM images of Ti_0.91_O_2_ nanosheets.

**Figure S8.** AFM images of Ti_0.87_Li_0.13_O_2_ nanosheets.

**Figure S9.** HAADF-STEM images of Ti_0.91_O_2_ nanosheets.

**Figure S10.** Enlarged HAADF-STEM image of **(a)** Ti_0.87_O_2_ nanosheets, and **(b)** the corresponding atomic column intensity map.

**Figure S11.** Enlarged HAADF-STEM image of **(a)** Ti_0.91_O_2_ nanosheets, and **(b)** the corresponding atomic column intensity map.

**Figure S12.** EPR spectroscopy **(a)** and calculation of g-factor **(b)** of Ti_0.87_O_2_, Ti_0.91_O_2_ and Ti_0.87_Li_0.13_O_2_ nanosheets. The g-factor proves the existence of Ti vacancies (1.985) and O vacancies (2.005) for both Ti_0.87_O_2_ and Ti_0.91_O_2_ nanosheets.^[1,2]^

**Figure S13.** High-resolution O 1s XPS spectra of Ti_0.87_O_2_, Ti_0.91_O_2_, Ti_0.87_Li_0.13_O_2_ and rutile-TiO_2_ (R-TiO_2_).

**Figure S14.** Zeta potentials of Ti_0.87_O_2_, Ti_0.91_O_2_, and Ti_0.87_Li_0.13_O_2_ nanosheets.

**Figure S15.** Illustration of changes in interlayer spacing during UV treatment of the Ti_0.87_O_2_ interfacial layer.

**Figure S16.** XRD pattern of original Ti_0.87_O_2_@Zn and after 2 hours of UV treatment.

**Figure S17.** XRD pattern of Ti_0.91_O_2_@Zn and Ti_0.87_Li_0.13_O_2_@Zn, with the interlayer spacing of the nanosheets estimated to be ~11.0 Å.

**Figure S18.** SAED pattern of the Ti_0.87_O_2_ interfacial layer.

**Figure S19.** The cross-sectional SEM images of Ti_0.87_O_2_@Zn with different thicknesses range from ~58 nm to 824 nm. **(a)** shows the SEM image of Ti_0.87_O_2_@Zn with a modification thickness of ~208 nm.

**Figure S20.** Relationship between mass loading and thickness for Ti_0.87_O_2_ layer.

**Figure S21.** SEM images showing the top-down view of **(a)** Ti_0.91_O_2_@Zn and **(b)** Ti_0.87_Li_0.13_O_2_@Zn.

**Figure S22.** Cross-sectional SEM images of **(a)** Ti_0.91_O_2_@Zn and **(b)** Ti_0.87_Li_0.13_O_2_@Zn.

**Figure S23.** Digital photographs of the Ti_0.87_O_2_@Zn under different conditions.

**Figure S24.** Zn^2+^ and SO_4_^2−^ concentrations in the side of deionized water with Ti_0.91_O_2_-PTFE and Ti_0.87_Li_0.13_O_2_-PTFE membrane during an 8h test.

**Figure S25.** Time-dependent pH changes of water separated from 0.5 M ZnSO_4_ by PTFE and Ti_0.87_O_2_–PTFE membranes, with air-exposed water as the control.

**Figure S26.** *I-V* curves of the Ti_0.87_O_2_-PTFE, Ti_0.91_O_2_-PTFE and Ti_0.87_Li_0.13_O_2_-PTFE recorded in ZnSO_4_ aqueous solutions at varying concentrations.

**Figure S27.** Interface models for 2 M ZnSO_4_ electrolyte and the stacked nanosheets **(a)** with dual confinement of interlayers and vacancies, **(b)** without confinement, **(c)** with single interlayer confinement, and **(d)** with single vacancy confinement.

**Figure S28.** Snapshots of the MD simulation taken at 100 ps for 2 M ZnSO_4_ electrolyte diffusion across the stacked nanosheets with single interlayer confinement.

**Figure S29.** Snapshots of the MD simulation taken at 100 ps for 2 M ZnSO_4_ electrolyte diffusion across the stacked nanosheets without confinement.

**Figure S30.** Nyquist plots of **(a)** Ti_0.87_O_2_@Zn, **(b)** Ti_0.91_O_2_@Zn, **(c)** Ti_0.87_Li_0.13_O_2_@Zn, and **(d)** bare Zn symmetrical cells at different temperatures based on EIS measurements.

**Figure S31.** Calculation of activation energy (E_a_) of Ti_0.87_O_2_@Zn, Ti_0.91_O_2_@Zn, Ti_0.87_Li_0.13_O_2_@Zn, and bare Zn anode based on Arrhenius plot fitting.

**Figure S32.** Raman spectrum of pure H_2_O and D_2_O.

**Figure S33. (a)** In situ Raman spectra of the solution in the upper chamber with PTFE as a barrier during an 8-hour static water permeation experiment, along with the corresponding (b) Raman intensity ratio of H_2_O to D_2_O.

**Figure S34.** Raman spectra of the O-H stretch vibration and corresponding signal ratios of O_1_, O_2_, O_3_, and O_4_ in pure water. O_1_ and O_2_ correspond to weakly hydrogen-bonded water species (W-HB), while O_3_ and O_4_ correspond to strongly hydrogen-bonded water species (S-HB).

**Figure S35.** Optimized theoretical simulation models of Ti_0.87_O_2_ **(a)**, Ti_0.91_O_2_ **(b)**, lepidocrocite TiO_2_ **(c),** and bare Zn **(d)**.

**Figure S36.** Calculated adsorption energies of Zn atom on the surfaces of Ti_0.87_O_2_, Ti_0.91_O_2_, L-TiO_2_, and bare Zn.

**Figure S37.** The ESP calculations of the Ti_0.87_O_2_ **(a)**, Ti_0.91_O_2_ **(b),** and lepidocrocite TiO_2_ **(c)**. (blue and red represent the negative and positive regions, respectively) 1 a.u. = 627.5095 kcal mol^−1^.

**Figure S38.** Zn^2+^ ion diffusion pathway in lepidocrocite TiO_2_ monolayer with Ti vacancy **(a, c)** and lepidocrocite TiO_2_ monolayer without Ti vacancy **(b, d)**.

**Figure S39.** Potential-energy curves of Zn^2+^ diffusion in Ti-deficient TiO_2_ and lepidocrocite TiO_2_ sheet.

**Figure S40.** The CV tests of (a) Ti_0.87_O_2_@Cu//Ti_0.87_O_2_@Zn, Ti_0.91_O_2_@Cu//Ti_0.91_O_2_@Zn, Ti_0.87_Li_0.13_O_2_@Cu//Ti_0.87_Li_0.13_O_2_@Zn, and Cu//Zn.

**Figure S41.** Zn^2+^ diffusion coefficient (*D_Zn_^2+^*) from the simplified Randles-Sercik curves.

**Figure S42.** GITT profile of (a) Ti_0.87_O_2_@Zn, (b) Ti_0.91_O_2_@Zn, (c) Ti_0.87_Li_0.13_O_2_@Zn, and (d) bare Zn symmetric cells at 1 mA cm^−2^.

**Figure S43.** CA curves of Ti_0.87_O_2_@Zn, Ti_0.91_O_2_@Zn, Ti_0.87_Li_0.13_O_2_@Zn, and bare Zn symmetric cells under an overpotential of −150 mV.

**Figure S44.** SEM images of cycled Ti_0.91_O_2_@Zn and Ti_0.87_Li_0.13_O_2_@Zn after 20 cycles at 1 mA cm^−2^ and 1 mAh cm^−2^. The inset shows digital photographs of the anodes after 20 cycles.

**Figure S45.** **(a, b)** Surface morphology of Ti_0.87_O_2_@Zn and the morphology of the deposited Zn beneath the Ti_0.87_O_2_ layer after 20 cycles at 1 mA cm^−2^ and 1 mAh cm^−2^.

**Figure S46.** Synchronized voltage profiles of the bare Zn and Ti_0.87_O_2_@Zn anodes recorded during the in-situ optical microscopy observation at 1 mA cm^−2^.

**Figure S47.** In-situ optical microscopy images of Ti_0.91_O_2_@Zn and Ti_0.87_Li_0.13_O_2_@Zn symmetric cell at 1 mA cm^−2^ at different times.

**Figure S48.** XRD patterns and corresponding relative peak-area fractions of the initial Zn foil, bare Zn after plating, and Ti_0.87_O_2_@Zn after plating.

**Figure S49.** **(a)**Operando EIS and **(b)** DRT evolution of bare Zn//bare Zn during a 48-hour resting test.

**Figure S50.** **(a)**Operando EIS and **(b)** DRT evolution of Ti_0.87_O_2_@Zn//Ti_0.87_O_2_@Zn during a 48-hour resting test.

**Figure S51.** Tafel curves **(a)** of Ti_0.87_O_2_@Zn, Ti_0.91_O_2_@Zn, Ti_0.87_Li_0.13_O_2_@Zn, and bare Zn. The comparison of corrosion currents **(b)** of Ti_0.87_O_2_@Zn, Ti_0.91_O_2_@Zn, Ti_0.87_Li_0.13_O_2_@Zn, and bare Zn.

**Figure S52.** LSV profiles of Ti_0.87_O_2_@Zn, Ti_0.91_O_2_@Zn, Ti_0.87_Li_0.13_O_2_@Zn, and bare Zn toward HER in 1 M Na_2_SO_4_ electrolyte.

**Figure S53.** XRD analysis of Ti_0.87_O_2_@Zn and bare Zn anodes after 50 cycles at a current density of 1 mA cm^−2^ and a capacity of 1 mAh cm^−2^.

**Figure S54.** XRD patterns of Ti_0.87_O_2_@Zn after different cycle numbers **(a)** in a symmetric cell and magnified views **(b)** of specific region in **(a)**.

**Figure S55.** EPR spectroscopy of the Ti_0.87_O_2_ coating layer before and after 50 cycles in a symmetric cell at 1 mA cm^−2^ and 1 mAh cm^−2^. The g-factor proves the existence of Ti vacancies (1.985) and O vacancies (2.005).

**Figure S56.** (a) XPS survey spectra, (b) high-resolution Ti 2p spectra, and (c) high-resolution O 1s spectra of the Ti_0.87_O_2_@Zn anode before and after 50 cycles at 1 mA cm⁻^2^ and 1 mAh cm⁻^2^.

**Figure S57.** Galvanostatic stripping/plating performance of symmetric cells with Zn anode modified with Ti_0.87_O_2_ interfacial layer with different thicknesses. The thicknesses of the Ti_0.87_O_2_ nanosheets used are **(a)** ~50 nm, **(b)** ~100 nm, **(c)** ~400 nm, and **(d)** ~800 nm, with a current density of 1 mA cm^−2^ and a capacity of 1 mAh cm^−2^.

**Figure S58.** Cycling performance of Ti_0.87_O_2_@Zn, Ti_0.91_O_2_@Zn, Ti_0.87_Li_0.13_O_2_@Zn, and bare Zn symmetric cells at 2 mA cm^−2^, 1 mAh cm^−2^.

**Figure S59.** Rate performance at various current densities with a capacity of 1 mAh cm^−2^ for Ti_0.87_O_2_@Zn, Ti_0.91_O_2_@Zn, Ti_0.87_Li_0.13_O_2_@Zn, and bare Zn symmetric cells.

**Figure S60.** Overpotential of Ti_0.87_O_2_@Zn, Ti_0.91_O_2_@Zn, Ti_0.87_Li_0.13_O_2_@Zn, and bare Zn symmetric cells at different current densities.

**Figure S61.** Intermittent galvanostatic charge/discharge performance of Ti_0.87_O_2_@Zn and bare Zn symmetric cells at 1 mA cm⁻^2^ and 1 mAh cm⁻^2^.

**Figure S62.** Depth-of-discharge cycling performance of Ti_0.87_O_2_@Zn and bare Zn symmetric cells using 20 μm Zn foil at 5 mA cm⁻^2^ and 10 mAh cm⁻^2^, corresponding to a Zn utilization of approximately 85.5% DOD.

**Figure S63.** Voltage profiles of Zn plating/stripping processes in **(a)** Ti_0.87_O_2_@Zn//Cu, **(b)** Ti_0.91_O_2_@Zn//Cu, **(c)** Ti_0.87_Li_0.13_O_2_@Zn//Cu, and **(d)** bare Zn//Cu cell at 1 mA cm^−2^ and 1 mAh cm^−2^.

**Figure S64.** **(a, b)** SEM images and **(c, b)** XRD patterns of NVO and VO_2_ cathode materials.

**Figure S65.** CV curves of **(a)** Ti_0.87_O_2_@Zn//NVO and **(b)** Ti_0.87_O_2_@Zn//VO_2_ at a scan rate of 0.1 mV s^−1^.

**Figure S66.** SEM images of Ti_0.87_O_2_@Zn **(a)** and bare Zn **(b)** after 50 cycles at a current density of 4 A g^−1^.

**Figure S67.** Self-discharge test of Ti_0.87_O_2_@Zn//VO_2_ and bare Zn//VO_2_ cells.

**Table S1. |** Comparison of the internal channel sizes and thicknesses of different materials modified on zinc anodes.

| **Materials** | **Morphology** | **Thickness (nm)** | **Channel size (Å)** | **Ref.** |
| --- | --- | --- | --- | --- |
| COF-Ti_6_Cu_3_ | nanosheets  (~ 4.8 nm) | ~ 2000 | ~23 | (3) |
| Fluorinated COF | film | 100 | ~20-30 | (4) |
| Sulfonate-rich COFs | nanoparticles | ~ 10000 | ＞10 | (5) |
| COF | film | ~ 70 | ＞10 | (6) |
| Fluorinated COF | film | ~ 100 | ＞10 | (7) |
| MOF-801 | nanoparticles | ~ 156000 | ~5.60 | (8) |
| Zif-7 | nanoparticles | - | 2.94 | (9) |
| MOF-5W | nanoparticles | ~ 15000 | ~7.80 | (10) |
| MOF-808 | nanoparticles | ~ 15000 | ~10.10 | (10) |
| Ti-based MOF | nanosheets  (~ 10 nm) | ~ 20000 | ~5-7 | (11) |
| Kaolin | nanoparticles | ~ 21000 | ~30 | (12) |
| OH-F-mSiO_2_ | film | ~ 100 | ＞10 | (13) |
| Ti_0.87_O_2_ | nanosheets  (~ 1.1 nm) | 200 | ~3.0 × 3.8 (in-plane)  ~3.5 (interlayer) | This Work |

**Table S2. |** Comparison of cycling performance for Ti_0.87_O_2_@Zn and reported modified Zn anodes.

| **Anodes** | **Thickness**  **(nm)** | **Current density**  **(mA cm**^−^**^2^)** | **Capacity**  **(mAh cm**^−^**^2^)** | **Cycling time**  **(h)** | **Ref.** |
| --- | --- | --- | --- | --- | --- |
| TpPa-SO_3_H@Zn | 100 | 1 | 5 | 1000 | (14) |
|  |  | 5 |  |  |  |
| Cu_3_N@Zn | 400 | 0.5 | 0.5 | 800 | (15) |
|  |  | 1 | 1 | 240 |  |
| ZnS@Zn | 500 | 2 | 2 | 1100 | (16) |
| ICOF@Zn | 360 | 1 | 1 | 1020 | (17) |
|  |  | 2 | 2 | 620 |  |
| ZnSiO_3_@Zn | 300 | 1 | 1 | 1600 | (18) |
|  |  | 5 |  | 1520 |  |
| Ag@Zn | 200 | 1 | 1 | 2070 | (19) |
|  |  | 2 | 1 | 2000 |  |
|  |  | 5 | 1.25 | 1580 |  |
| MXene@Zn | 150 | 0.2 | 0.2 | 820 | (20) |
|  |  | 0.5 | 1 | 350 |  |
|  |  | 1 | 1 | 150 |  |
| FCOF@Zn | 100 | 5 | 1 | 1700 | (4) |
| BN@Zn | 100 | 1 | 1 | 3000 | (21) |
|  |  | 5 | 2.5 | 1600 |  |
| rGo@Zn | 100 | 1 | 2 | 200 | (22) |
|  |  | 2 |  |  |  |
|  |  | 5 |  |  |  |
| MoS_2_@Zn | 70 | 2.5 | 0.416 | 170 | (23) |
| HsGDY@Zn | 490 | 0.5 | 0.5 | 2400 | (24) |
|  |  | 1 | 1 | 2400 |  |
| ZnO:S@Zn | 200 | 0.5 | 1 | 1420 | (25) |
|  |  | 1 |  | 1680 |  |
| β-PVDF@Zn | 200 | 0.25 | 0.05 | 2000 | (26) |
|  |  | 1.5 | 0.3 | 100 |  |
| NGo@Zn | 120 | 1 | 1 | 1200 | (27) |
|  |  | 5 | 5 | 300 |  |
| Ti_0.87_O_2_@Zn | 200 | 1 | 1 | 5000 | This work |
|  |  | 2 |  | 4400 |  |
|  |  | 5 |  | 4000 |  |

**Table S3. |** Comparison of capacity retention of full cells with different Zn anode.

| **Anodes** | **Cathode** | **Current density**  **(A g**^−^**^1^)** | **Capacity decay per cycle** | **Ref.** |
| --- | --- | --- | --- | --- |
| NGo@Zn | LiMn_2_O_4_ | 0.148 | ~0.112% | (27) |
| Polyamide@Zn | α-MnO_2_ | 0.6 | ~0.0217% | (28) |
| COF-DIP@Zn | δ-MnO_2_ | 2 | ~0.0383% | (6) |
| indium-carbon@Zn | MnO_2_ | 1 | ~0.0109% | (29) |
| SCOFs@Zn | α-MnO_2_ | 1 | ~0.0792% | (5) |
| PT-ZHC-Sn@Zn | MnO_2_ | 2 | ~0.0178% | (30) |
| MOF-801@Zn | NH_4_V_4_O_10_ | 5 | ~0.0068% | (8) |
| polydopamine@Zn | MnO_2_ | 0.5 | ~0.0313% | (31) |
| MPVMT@Zn | MnO_2_ | ~0.6 | ~0.0594% | (32) |
| hydrogel@Zn | NaV_3_O_8_·1.5H_2_O | 5 | ~0.0067% | (33) |
| Cu-MXene-Zn | NaV_3_O_8_·1.5H_2_O | 4 | ~0.0237% | (34) |
| MXene@Zn | MnO_2_ | 1 | ~0.0380% | (20) |
| MoS_2_-Zn | α-MnO_2_ | 1 | ~0.0185% | (23) |
| kaolin@Zn | MnO_2_ | 2 | ~0.0197% | (12) |
| CD-MWCNT@Zn | V_2_O_5_ | 1 | ~0.04375% | (35) |
| Zn(O_3_PCH_2_OH)@Zn | NaV_3_O_8_·1.5H_2_O | 20 | ~0.016% | (36) |
| ZnSiO_3_@Zn | K_0.27_MnO_2_·0.54H_2_O | 0.308 | ~0.0735% | (18) |
| HEO-CNFs@Zn | NH_4_V_4_O_10_ | 10 | ~0.0058% | (37) |
| CN10@Zn | Na_0.65_Mn_2_O_4_ | 2 | ~0.011% | (38) |
| ZnO@Zn | V_2_O_5_ | 1 | ~0.026% | (39) |
| NGo@Zn | LiMn_2_O_4_ | 0.148 | ~0.112% | (27) |
| (CS/SA)_4_@Zn | H_2_V_3_O_8_ | 0.034 | ~0.034% | (40) |
| Ti_0.87_O_2_@Zn | NaV_3_O_8_·1.5H_2_O | 4 | ~0.0056% | This work |
| Ti_0.87_O_2_@Zn | VO_2_ | 3 | ~0.0070% | This work |

References

[1]	S. M. Wu, X. L. Liu, X. L. Lian, G. Tian, C. Janiak, Y. X. Zhang, Y. Lu, H. Z. Yu, J. Hu, H. Wei, H. Zhao, G. G. Chang, G. Van Tendeloo, L. Y. Wang, X. Y. Yang, B. L. Su, Homojunction of oxygen and titanium vacancies and its interfacial N-P effect. *Adv. Mater.* **30**, 1802173 (2018).

[2]	G. Luo, D. Liu, J. Zhao, A. Hussain, W. Raza, Y. Wu, F. Liu, X. Cai, Negatively charged holey titania nanosheets added electrolyte to realize dendrite-free lithium metal battery. *Small* **19**, 2206176 (2023).

[3]	C. Guo, J. Zhou, Y. Chen, H. Zhuang, J. Li, J. Huang, Y. Zhang, Y. Chen, S. L. Li, Y. Q. Lan, Integrated micro space electrostatic field in aqueous Zn-ion battery: Scalable electrospray fabrication of porous crystalline anode coating. *Angew. Chem. Int. Ed.* **62**, 202300125 (2023).

[4]	Z. Zhao, R. Wang, C. Peng, W. Chen, T. Wu, B. Hu, W. Weng, Y. Yao, J. Zeng, Z. Chen, P. Liu, Y. Liu, G. Li, J. Guo, H. Lu, Z. Guo, Horizontally arranged zinc platelet electrodeposits modulated by fluorinated covalent organic framework film for high-rate and durable aqueous zinc ion batteries. *Nat. Commun.* **12**, 6606 (2021).

[5]	Y. Wang, Z. Deng, B. Luo, G. Duan, S. Zheng, L. Sun, Z. Ye, J. Lu, J. Huang, Y. Lu, Highly reversible Zn metal anodes realized by synergistically enhancing ion migration kinetics and regulating surface energy. *Adv. Funct. Mater.* **32**, 2209028 (2022).

[6]	J. H. Park, M. J. Kwak, C. Hwang, K. N. Kang, N. Liu, J. H. Jang, B. A. Grzybowski, Self-assembling films of covalent organic frameworks enable long-term, efficient cycling of zinc-ion batteries. *Adv. Mater.* **33**, 2101726 (2021).

[7]	D. Lei, W. Shang, L. Cheng, Poonam, W. Kaiser, P. Banerjee, S. Tu, O. Henrotte, J. Zhang, A. Gagliardi, J. Jinschek, E. Cortés, P. Müller‐Buschbaum, A. S. Bandarenka, M. Z. Hussain, R. A. Fischer, Ion‐transport kinetics and interface stability augmentation of zinc anodes based on fluorinated covalent organic framework thin films. *Adv. Energy Mater.* **14**, 2403030 (2024).

[8]	D. Xu, Z. Wang, C. Liu, H. Li, F. Ouyang, B. Chen, W. Li, X. Ren, L. Bai, Z. Chang, A. Pan, H. Zhou, Water catchers within sub-nano channels promote step-by-step zinc-ion dehydration enable highly efficient aqueous zinc-metal batteries. *Adv. Mater.* **36**, 2403765 (2024).

[9]	H. Yang, Z. Chang, Y. Qiao, H. Deng, X. Mu, P. He, H. Zhou, Constructing a super-saturated electrolyte front surface for stable rechargeable aqueous zinc batteries. *Angew. Chem. Int. Ed.* **59**, 9377-9381 (2020).

[10]	W. Zhang, W. Qi, K. Yang, Y. Hu, F. Jiang, W. Liu, L. Du, Z. Yan, J. Sun, Boosting tough metal Zn anode by MOF layer for high-performance zinc-ion batteries. *Energy Storage Mater.* **71**, 103616 (2024).

[11]	C. Wang, Q. Xie, T. Guo, M. Fang, W. Mao, Y. Zhang, H. Wang, X. Ma, Y. Wu, S. Li, J. Han, Understanding the role of titanium metal-organic framework nanosheets in modulating anode chemistry for aqueous zinc-ion batteries. *Nano Lett.* **23**, 10930-10938 (2023).

[12]	C. Deng, X. Xie, J. Han, Y. Tang, J. Gao, C. Liu, X. Shi, J. Zhou, S. Liang, A sieve‐functional and uniform‐porous kaolin layer toward stable zinc metal anode. *Adv. Funct. Mater.* **30**, 2000599 (2020).

[13]	L. Wang, B. Zhang, W. Zhou, Z. Zhao, X. Liu, R. Zhao, Z. Sun, H. Li, X. Wang, T. Zhang, H. Jin, W. Li, A. Elzatahry, Y. Hassan, H. J. Fan, D. Zhao, D. Chao, Tandem chemistry with Janus mesopores accelerator for efficient aqueous batteries. *J. Am. Chem. Soc.* **146**, 6199-6208 (2024).

[14]	J. Zhao, Y. Ying, G. Wang, K. Hu, Y. D. Yuan, H. Ye, Z. Liu, J. Y. Lee, D. Zhao, Covalent organic framework film protected zinc anode for highly stable rechargeable aqueous zinc-ion batteries. *Energy Storage Mater.* **48**, 82-89 (2022).

[15]	Z. Yang, C. Lv, W. Li, T. Wu, Q. Zhang, Y. Tang, M. Shao, H. Wang, Revealing the two-dimensional surface diffusion mechanism for zinc dendrite formation on zinc anode. *Small* **18**, 2104148 (2022).

[16]	J. Hao, B. Li, X. Li, X. Zeng, S. Zhang, F. Yang, S. Liu, D. Li, C. Wu, Z. Guo, An in-depth study of Zn metal surface chemistry for advanced aqueous Zn-ion batteries. *Adv. Mater.* **32**, 2003021 (2020).

[17]	L. Yang, Q. Ma, Y. Yin, D. Luo, Y. Shen, H. Dou, N. Zhu, R. Feng, Y. Kong, A. Yu, B. Cheng, X. Wang, Z. Chen, Construction of desolvated ionic COF artificial SEI layer stabilized Zn metal anode by in-situ electrophoretic deposition. *Nano Energy* **117**, 108799 (2023).

[18]	R. Guo, X. Liu, F. Xia, Y. Jiang, H. Zhang, M. Huang, C. Niu, J. Wu, Y. Zhao, X. Wang, C. Han, L. Mai, Large-scale integration of a zinc metasilicate interface layer guiding well-regulated Zn deposition. *Adv. Mater.* **34**, 2202188 (2022).

[19]	Y. Zhang, K. Yan, Y. Jiang, S.-D. Liu, G.-X. Liu, Long-life and highly stable Zn metal anodes achieved by Ag nano-coating via electron beam evaporation. *Chem. Eng. J.* **502**, 157933 (2024).

[20]	N. Zhang, S. Huang, Z. Yuan, J. Zhu, Z. Zhao & Z. Niu, Direct self-assembly of MXene on Zn anodes for dendrite-free aqueous zinc-ion batteries. *Angew. Chem. Int. Ed.* **60**, 2861-2865 (2021).

[21]	H. Jia, M. Qiu, C. Tang, H. Liu, S. Fu, X. Zhang, Nano‐scale BN interface for ultra‐stable and wide temperature range tolerable Zn anode. *EcoMat* **4**, 12190 (2022).

[22]	C. Shen, X. Li, N. Li, K. Xie, J. G. Wang, X. Liu, B. Wei, Graphene-boosted, high-performance aqueous Zn-ion battery. *ACS Appl. Mater. Interfaces* **10**, 25446-25453 (2018).

[23]	S. Bhoyate, S. Mhin, J. E. Jeon, K. Park, J. Kim, W. Choi, Stable and high-energy-density Zn-ion rechargeable batteries based on a MoS_2_-coated Zn anode. *ACS Appl. Mater. Interfaces* **12**, 27249-27257 (2020).

[24]	Q. Yang, Y. Guo, B. Yan, C. Wang, Z. Liu, Z. Huang, Y. Wang, Y. Li, H. Li, L. Song, J. Fan, C. Zhi, Hydrogen-substituted graphdiyne ion tunnels directing concentration redistribution for commercial-grade dendrite-free zinc anodes. *Adv. Mater.* **32**, 2001755 (2020).

[25]	D. Zhu, Y. Zheng, Y. Xiong, C. Cui, F. Ren, Y. Liu, In situ growth of S-doped ZnO thin film enabling dendrite-free zinc anode for high-performance aqueous zinc-ion batteries. *J. Alloys Compd.* **918**, 165486 (2022).

[26]	L. T. Hieu, S. So, I. T. Kim & J. Hur, Zn anode with flexible β-PVDF coating for aqueous Zn-ion batteries with long cycle life. *Chem. Eng. J.* **411**, 128584 (2021).

[27]	J. Zhou, M. Xie, F. Wu, Y. Mei, Y. Hao, R. Huang, G. Wei, A. Liu, L. Li, R. Chen, Ultrathin surface coating of nitrogen-doped graphene enables stable zinc anodes for aqueous zinc-ion batteries. *Adv. Mater.* **33**, 2101649 (2021).

[28]	Z. Zhao, J. Zhao, Z. Hu, J. Li, J. Li, Y. Zhang, C. Wang, G. Cui, Long-life and deeply rechargeable aqueous Zn anodes enabled by a multifunctional brightener-inspired interphase. *Energy Environ. Sci.* **12**, 1938-1949 (2019).

[29]	J. Yin, Y. Wang, Y. Zhu, J. Jin, C. Chen, Y. Yuan, Z. Bayhan, N. Salah, N. A. Alhebshi, W. Zhang, U. Schwingenschlögl, H. N. Alshareef, Regulating the redox reversibility of zinc anode toward stable aqueous zinc batteries. *Nano Energy* **99**, 107331 (2022).

[30]	Z. Shao, L. Lin, W. Zhuang, S. Liu, P. Yang, K. Zhu, C. Li, G. Guo, W. Wang, Q. Zhang, Y. Yao, In situ self-reconfiguration induced multifunctional triple-gradient artificial interfacial layer toward long-life Zn-metal anodes. *Adv. Mater.* **36**, 2406093 (2024).

[31]	T. Wang, P. Wang, L. Pan, Z. He, L. Dai, L. Wang, S. Liu, S. C. Jun, B. Lu, S. Liang, J. Zhou, Stabling zinc metal anode with polydopamine regulation through dual effects of fast desolvation and ion confinement. *Adv. Energy Mater.* **13**, 2203523 (2022).

[32]	Z. Zheng, X. Zhong, Q. Zhang, M. Zhang, L. Dai, X. Xiao, J. Xu, M. Jiao, B. Wang, H. Li, Y. Jia, R. Mao, G. Zhou, An extended substrate screening strategy enabling a low lattice mismatch for highly reversible zinc anodes. *Nat. Commun.* **15**, 753 (2024).

[33]	J. L. Yang, J. Li, J. W. Zhao, K. Liu, P. Yang, H. J. Fan, Stable zinc anodes enabled by a zincophilic polyanionic hydrogel layer. *Adv. Mater.* **34**, 2202382 (2022).

[34]	Y. Li, Q. Zhu, M. Xu, B. Zang, Y. Wang, B. Xu, Cu‐modified Ti_3_C_2_Cl_2_ MXene with zincophilic and hydrophobic characteristics as a protective coating for highly stable Zn anode. *Adv. Funct. Mater.* **33**, 2213416 (2023).

[35]	L. Kang, K. Yue, C. Ma, H. Yuan, J. Luo, Y. Wang, Y. Liu, J. Nai, X. Tao, Mediating Zn ions migration behavior via beta-cyclodextrin modified carbon nanotube film for high-performance Zn anodes. *Nano Lett.* **24**, 4150-4157 (2024).

[36]	J. He, Y. Tang, G. Liu, H. Li, M. Ye, Y. Zhang, Q. Yang, X. Liu, C. Li, Intrinsic hydrogen‐bond donors‐lined organophosphate superionic nanochannels levering high‐rate‐endurable aqueous Zn batteries. *Adv. Energy Mater.* **12**, 2202661 (2022).

[37]	Y. Li, H. Jia, U. Ali, H. Wang, B. Liu, L. Li, L. Zhang, C. Wang, Successive gradient internal electric field strategy toward dendrite‐free zinc metal anode. *Adv. Energy Mater.* **13**, 2301643 (2023).

[38]	F. Zhao, J. Feng, H. Dong, R. Chen, T. Munshi, I. Scowen, S. Guan, Y. E. Miao, T. Liu, I. P. Parkin, G. He, Ultrathin protection layer via rapid sputtering strategy for stable aqueous zinc ion batteries. *Adv. Funct. Mater.* **16**, 2409400 (2024).

[39]	X. Hu, J. Borowiec, Y. Zhu, X. Liu, R. Wu, A. M. Ganose, I. P. Parkin, B. D. Boruah, Dendrite-free zinc anodes enabled by exploring polar-face-rich 2D ZnO interfacial layers for rechargeable Zn-ion batteries. *Small* **20**, 2306827 (2024).

[40]	X. Cai, X. Wang, Z. Bie, Z. Jiao, Y. Li, W. Yan, H. J. Fan, W. Song, A layer-by-layer self-assembled bio-macromolecule film for stable zinc anode. *Adv. Mater.* **36**, 2306734 (2024).
